# Photocatalytic degradation and transformation of pharmaceuticals using exfoliated metal-free g-C_3_N_4_

**DOI:** 10.1016/j.isci.2025.113899

**Published:** 2025-10-30

**Authors:** Petr Praus, Anna Gavlová, Jan Hrbáč, Kristina Schmidtová, Petr Bednář

**Affiliations:** 1 Institute of Environmental Technology, CEET, VSB-Technical University of Ostrava, 17. Listopadu 15, Ostrava 70800, Czech Republic; 2 Department of Chemistry, Faculty of Science, University of Ostrava, 30. Dubna 22, Ostrava 70103, Czech Republic; 3 Department of Analytical Chemistry, Faculty of Science, Palacky University, 17. Listopadu 12, Olomouc 77900, Czech Republic; 4 Institute of Chemistry, Faculty of Science, Masaryk University, Kamenice 5, Brno 62000, Czech Republic; 5 Department of Chemistry and Physico-Chemical Processes, Faculty of Materials Science and Technology, VSB-Technical University of Ostrava, 17. Listopadu 15, Ostrava 70800, Czech Republic

**Keywords:** Drugs, Chemical reaction, Catalysis, Environmental Chemical Engineering

## Abstract

Pharmaceuticals are micropollutants of global concern that contribute to environmental contamination alongside other anthropogenic and natural chemical compounds. This study addresses the photocatalytic degradation of model pharmaceutical compounds ofloxacin, diclofenac, and caffeine using bulk and thermally exfoliated graphitic carbon nitride (g-C_3_N_4_). Bulk g-C_3_N_4_ was synthesized from dicyandiamide at 550°C and exfoliated at 500 °C for 1-3 h in an ambient atmosphere. The structural, textural, and electronic properties of the prepared materials were evaluated.

Graphitic carbon nitride exfoliated for 2 h provided the best photocatalytic degradation efficiencies (>95%) for both ofloxacin and diclofenac and approximately 80% for caffeine, determined for 120 min under irradiation at 420 nm. The pharmaceuticals were degraded, and their intermediate degradation products were investigated using liquid chromatography combined with high-resolution tandem mass spectrometry. The successful identification of the main degradation products allowed us to propose transformation pathways for the studied pharmaceuticals.

## Highlights

• Photocatalysis enables 80–98% degradation of selected pharmaceuticals within 120 min
• g-C_3_N_4_ thermally exfoliated for 2 h is an optimal photocatalyst under visible light
• Degradation products and transformation pathways are identified by mass spectrometry

## Graphical abstract

**Figure:**
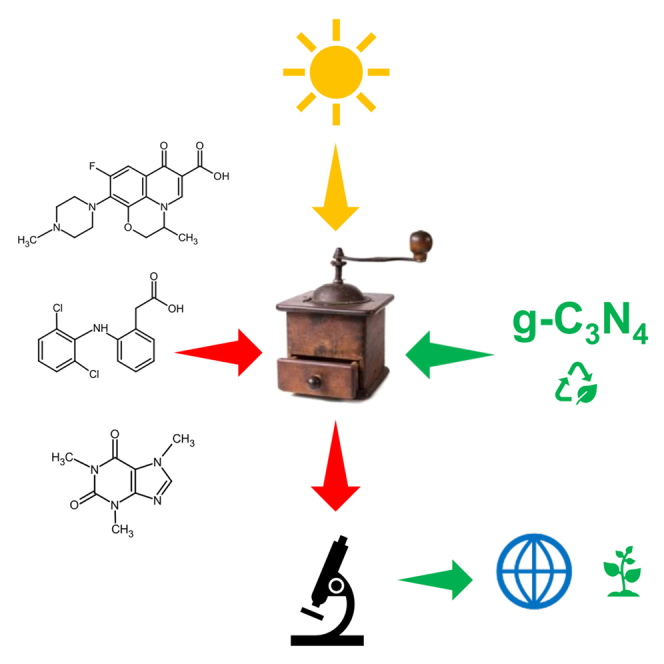

## Introduction

Pharmaceuticals of both anthropogenic and natural origins contribute to the growing issue of micropollutant contamination worldwide. These substances can enter aquatic systems through various means, including the excretion of non-metabolized drugs by humans and animals, improper disposal of unused medications, and industrial and agricultural runoff. Urban wastewater treatment plants are significant point sources, as they often fail to completely remove these compounds during treatment processes. Pharmaceuticals have been found in surface water, groundwater, soil, and sediments. In addition to the complexity of these samples, they often exist in trace concentrations at the level of nanogrammes or micrograms per liter or kilogram in liquid or solid samples of aquatic systems, which makes their analytical determination difficult.

Traditional wastewater treatment based on the activated sludge process is only partially effective in removing pharmaceuticals.^1^^,^^2^ In principle, adsorption and separation processes retain pharmaceuticals; however, they do not address the ultimate disposal of the separated organic compounds. In contrast, photocatalysis enables the removal of pharmaceuticals from aqueous environments by their degradation.^3^ Photocatalysis is part of advanced oxidation processes (AOPs) that can be used to treat organic pollutants in water. AOPs are usually performed using ozone, hydrogen peroxide, persulphate, peroxymonosulfate, ozone, sonolysis, and iron salts in the Fenton process.^4^ Photocatalysis has also been used for various reactions, such as the reduction of CO_2_,^5^ degradation of dyes^6^ and other organic compounds,^7^ hydrogen evolution by water splitting^8^ or adding NaBH_4_,^9^ fixation of nitrogen,^10^ reduction of Cr(VI),^11^ synthesis of hydrogen peroxide,^12^ water disinfection,^13^ and gene removal.^14^ The application of Fe-MOF-based composites is also a promising strategy for the photocatalytic removal of environmental pollutants.^15^

Most photocatalytic applications are based on TiO_2_, but graphitic carbon nitride has also been found to be a suitable photocatalyst for the degradation of various organic compounds, including pharmaceuticals. This material has been investigated over the last decade because it can be activated by visible irradiation owing to its narrow band gap (2.7 eV) and diamond-like physicochemical properties, such as mechanical, thermal, and chemical stability.^16^ The popularity of g-C_3_N_4_ also lies in its simple preparation via thermal synthesis from nitrogen-rich organic precursors, such as cyanamide, dicyandiamide, and melamine, in relatively high quantities at a low cost.

However, the low specific surface area and fast recombination of photoinduced electron - hole pairs, resulting in low quantum efficiency, are drawbacks of g-C_3_N_4_. The low specific surface area of pre-prepared (bulk) g-C_3_N_4_ can be increased by exfoliation using various methods, such as chemical oxidation with K_2_Cr_2_O_7_, concentrated sulfuric acid, organic liquids, sonication, ion intercalation, and thermal exfoliation. Thermal exfoliation, notable for its simplicity, ease of implementation, and environmental friendliness, is often used. In addition, the physico-chemical properties of g-C_3_N_4_ can be fine-tuned by coupling with anions, metal cations, metal nanoparticles such as Au and Au/Pd, Ag, Cu, Cu/Co, or semiconductor particles such as TiO_2_, ZnO, FeS_2_, Al_2_O_3_, SnO_2_, WO_3_, Ag_3_VO_4_, BiVO_4_, BiOBr, BiIO_4_, and Cu_3_V_2_O_8_. Another approach is doping with metal and non-metal elements, such as O, S, and P.

This study aimed to investigate the photocatalytic degradation of pharmaceuticals, including their transformation products. For this purpose, bulk and thermally exfoliated g-C_3_N_4_ samples were used in this study. This is a simple, one-component, metal-free, and low-cost photocatalyst with potential application in water treatment technology. Moreover, its photocatalytic stability during the degradation of organic substances has been reported in the literature.^17^ The photocatalysts were synthesized from dicyandiamide and characterized by elemental analysis, electron microscopy, X-ray diffraction, physisorption of nitrogen, and infrared, UV-Vis, photoluminescence, and electron paramagnetic resonance spectroscopies. In photocatalytic experiments, the common and widespread pharmaceuticals ofloxacin, diclofenac, and caffeine, selected as model compounds, were degraded under visible light irradiation at 420 nm. The degradation rates and efficiencies were evaluated, and degradation pathways were suggested based on the identification of intermediary degradation products using liquid chromatography combined with high-resolution tandem mass spectrometry.

The novelty of this study lies in the investigation of the transformation pathways of selected pharmaceuticals, such as ofloxacin, diclofenac, and caffeine. Such transformation investigations are important for possible water treatment applications, but are often omitted from reported studies. An important feature is that pure g-C_3_N_4_ can be an effective and suitable photocatalyst, which can be simply tuned through thermal exfoliation to achieve results comparable to those of other complex multi-component photocatalytic systems. Moreover, unlike other photocatalysts, such as TiO_2_, g-C_3_N_4_ operates under visible light irradiation.

## Results and discussion

The g-C_3_N_4_ bulk and exfoliated materials were synthesized from dicyandiamide in air, and their physico-chemical properties were extensively characterized. The photocatalytic properties were studied by the degradation of selected pharmaceuticals (ofloxacin, diclofenac, and caffeine), and the degradation products were analyzed by liquid chromatography. Transformation pathways were proposed for all three pharmaceuticals studied.

### Elemental analysis

The synthesized bulk and exfoliated g-C_3_N_4_ samples were analyzed to determine their elemental compositions (Table 1). The C, H, and N contents were determined directly, while the O content was calculated by difference to reach a total of 100%. The C and N contents were similar in the exfoliated g-C_3_N_4_ samples but were generally lower than those in bulk g-C_3_N_4_ because these elements were released during the exfoliation process, indicating the formation of both nitrogen and carbon defects (vacancies). The oxygen and hydrogen contents were higher in the exfoliated materials owing to their partial oxidation in air and the formation of -OH groups, as shown in the Fourier transform infrared (FTIR) spectra (Figure 4).

**Table 1:** Elemental composition of bulk and exfoliated g-C_3_N_4_

Material	C (wt. %)	H (wt. %)	N (wt. %)	C/N	O (wt. %)
Bulk	35.11	1.53	62.40	0.563	0.96
TEX1	34.35	1.85	61.40	0.559	2.40
TEX2	34.45	1.80	61.73	0.558	2.02
TEX3	34.25	1.83	61.33	0.558	2.59

### Characterization by electron microscopy

**Figure 1:**
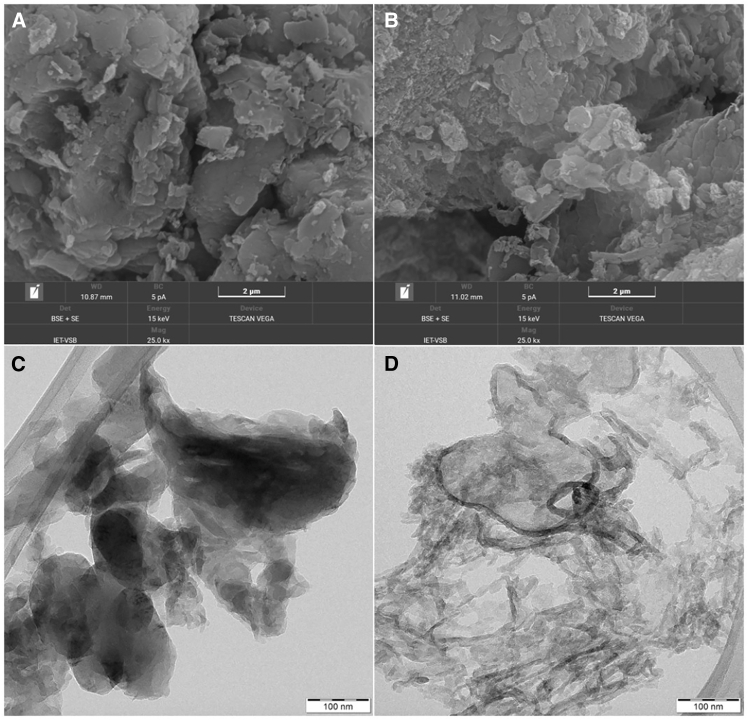
Characterization by electron microscopy SEM (BSE+SE) micrographs of (A) Bulk and (B) TEX3 graphitic carbon nitride. TEM micrographs of (C) Bulk and (D) TEX3 graphitic carbon nitride. SEM analysis: the acceleration voltage was 30 keV. The samples were gold-sputtered before analysis to ensure adequate electron conductivity. TEM analysis: an accelerating voltage of 200 kV was applied. Ethanol dispersions were placed on copper grids with holey carbon films.

The morphologies of the g-C_3_N_4_ materials were investigated by scanning electron microscopy (SEM). Micrographs of the Bulk and TEX3 samples (samples with the lowest and highest surface areas, respectively) are presented in Figure 1. Their morphologies were very similar, that is, large particles composed of aggregated flakes. The results of the SEM-EDS analysis are summarized in Table S1. The nitrogen, carbon, and oxygen contents of g-C_3_N_4_, as well as the C/N ratios, do not correspond with the results of the elemental analysis given in Table 1. The EDS results are not realistic for several reasons, namely a small amount of the analyzed materials, the impossibility to determine hydrogen, and a low sensitivity of EDS to light elements (C, N). The data can also be distorted due to the use of carbon tape to affix the powder samples. Therefore, we consider the results of conventional elemental analysis, which uses at least 0.5 g of each sample, to be reliable and realistic. However, a useful piece of information from the SEM-EDS is that the synthesized g-C_3_N_4_ materials were not contaminated by other elements.

The transmission electron microscopy (TEM) micrographs are shown in Figure 1. The presence of worm-like structures is typical of exfoliated g-C_3_N_4_ materials. One can also see large flat flakes with different thicknesses and agglomerations. The worm-like structures were probably formed by the wrapping and deformation of flat flakes due to prolonged exposure to high temperatures.

### Characterization by X-Ray diffraction

**Figure 2:**
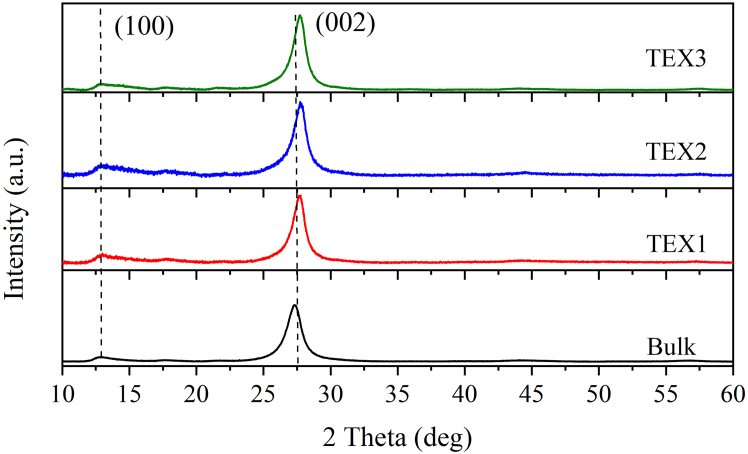
Characterization by X-ray diffraction XRD patterns of bulk and exfoliated g-C_3_N_4_ using a Cu tube operated at 40 kV and 30 mA.

The structures of the synthesized bulk and exfoliated g-C_3_N_4_ were studied using X-ray diffraction (XRD) (Figure 2). Two typical main diffraction peaks, (002) and (100), were observed (JCPDS 87–1526, supplemental information). The (002) and (100) diffractions can be ascribed to the interlayer stacking of the g-C_3_N_4_ planes and the in-plane ordering of the nitrogen-linked heptazine units, respectively. The selected characteristics of the diffraction peaks are listed in Table 2.

**Table 2:** XRD characteristics of bulk and exfoliated g-C_3_N_4_

Material	2θ (deg)	FWHM (deg)	L(002) (nm)	d(002) (nm)
Bulk	27.33	1.30	6.11	0.326
TEX1	27.73	1.08	7.36	0.321
TEX2	27.79	1.15	6.91	0.321
TEX3	27.72	1.10	7.22	0.322

Note: The 2θ values correspond to the (002) diffraction.

The d(002) spacing of bulk g-C_3_N_4_ decreased slightly after exfoliation, while the crystallite size L(002) increased. These structural changes can be explained by the formation of expanded crystallites owing to the thermal treatment of bulk g-C_3_N_4_.

### Characterization by X-Ray photoelectron spectroscopy

**Figure 3:**
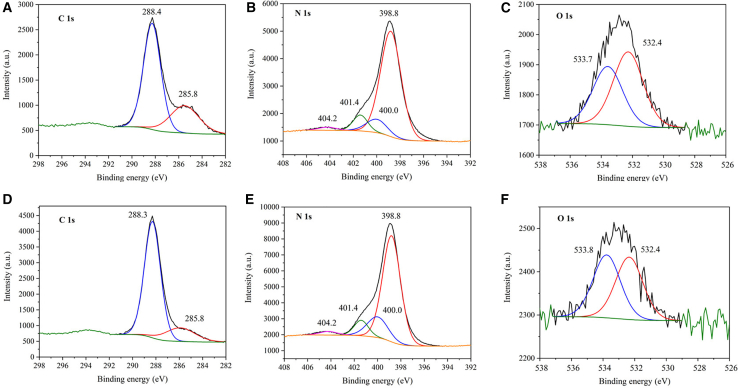
Characterization by X-ray photoelectron spectroscopy XPS spectra of (A–C) Bulk and (D–F) TEX3 g-C_3_N_4_ using Mg Kα radiation (hν = 1253.6 eV) generated at 12 kV and 10 mA.

X-ray photoelectron spectroscopy (XPS) was performed to investigate the surface composition of graphitic carbon nitride materials, specifically focusing on the bulk and TEX3 samples. The elemental compositions are summarized in Table S2. The C 1s spectra, shown in Figure 3, were deconvoluted into two main peaks with comparable binding energies: 285.6 and 288.4 eV for bulk and 285.8 and 288.3 eV for TEX3. These peaks comprised overlapping contributions from C–C (284.8 eV), C–N (∼286 eV), and C–O (286–287 eV) bonds. The peaks at 288.4 and 288.3 eV can be attributed to sp^2^-hybridized carbon atoms in N–C=N environments.^18^ The contributions from C–O and C–C bonding may arise from oxidative alterations and/or surface contamination by adventitious carbon. The C 1s spectra of the Bulk and TEX3 films exhibited differences, indicating a lower surface C content in TEX3 (Table S2) owing to the release of CO_2_ during thermal exfoliation.

The N 1s spectra (Figure 3) were fitted with four components located at 398.8, 400.0, 401.4, and 404.2 eV. The peaks at 398.8 eV and 400.0 eV correspond to sp^2^-hybridized nitrogen (N_2_C) and tertiary nitrogen (N_3_C), respectively, which are commonly associated with triazine units and amine-type linkages.^19^ The component at 401.4 eV is indicative of N atoms involved in C–N–H bonding configurations.^19^ Peaks at 404.2 and 404.3 eV may originate from π–π^∗^ (HOMO–LUMO) transitions^18^ or, alternatively, from surface charging effects.^19^ No significant spectral differences in the N 1s region were observed between the Bulk and TEX3.

In the O 1s spectra (Figure 3), the distinction between C–O and N–O, or C=O and N=O bonds, could not be reliably resolved. The broad peak was deconvoluted into two components: one at 532.4 eV, associated with C=O (or N=O) species, and another at 533.7 and 533.8 eV, attributed to C–O (or N–O) bonding environments. Because carbon and nitrogen have similar electronegativity values, the oxygen-binding site cannot be conclusively identified. The lower O surface content in TEX3 can also be explained by the release of CO_2_.

### Textural properties by nitrogen physisorption

**Figure 4:**
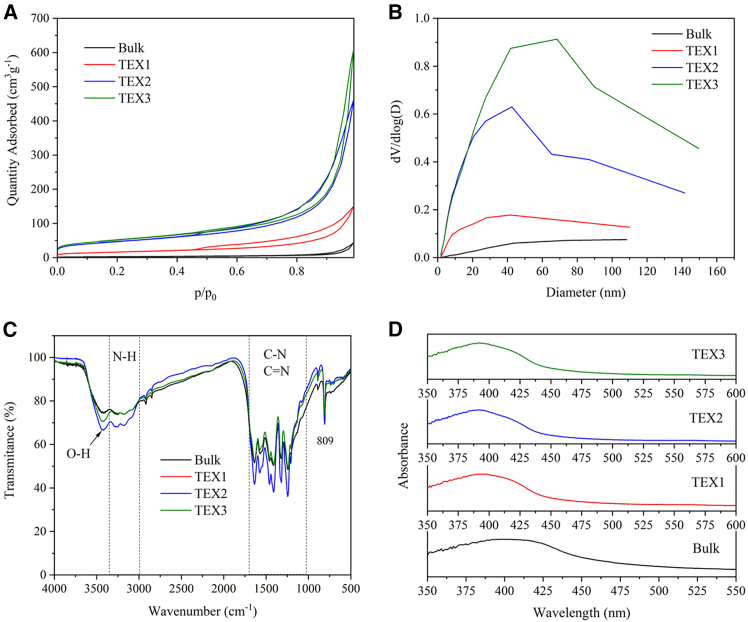
Textural and spectral characterization (A) Nitrogen adsorption and desorption isotherms at 77 K for bulk and exfoliated g-C_3_N_4_ samples. (B) The nitrogen adsorption–desorption data were processed according to the classical BET theory (for p/p_0_ ≈ 0.05–0.25). Distribution of pore sizes calculated using the BJH model. (C) FTIR spectra of bulk and exfoliated g-C_3_N_4_ with a resolution of 2 cm^−1^. The KBr method was employed. (D) UV-Vis absorption spectra of bulk and exfoliated g-C_3_N_4_ recorded using UV-Vis diffuse reflectance spectroscopy.

The textural properties of the bulk and exfoliated g-C_3_N_4_ samples were investigated through the physisorption of nitrogen. The adsorption–desorption isotherms are presented in Figure 4. All isotherms exhibited hysteresis loops, indicating the presence of mesopores in the materials. The pore size distributions were calculated according to the BJH model applied to the adsorption branch of the nitrogen adsorption-desorption isotherm. From the distribution curves, it is evident that mesopores dominate in the exfoliated samples (Figure 4). It can be concluded that the exfoliation of bulk g-C_3_N_4_ is accompanied by mesopore formation, leading to an increase in the specific surface area.

The specific surface area calculated using the BET method, surface area of the micropores, and pore volumes for the bulk and exfoliated g-C_3_N_4_ samples are summarized in Table 3. The S_BET_ increased with exfoliation time. A higher specific surface area of the catalyst provides more active sites for the adsorption of reactant molecules. The pore structure of semiconductors affects their photocatalytic activity owing to the larger contact interface and mass transfer.^20^ Moreover, there is a quantitative nonlinear relationship showing that the overall reaction rate increases with an increase in S_BET_ and the lifetime.^21^

**Table 3:** Textural characteristics of bulk and exfoliated g-C_3_N_4_

Material	S_BET_ (m^2^ g^−1^)	S_m_(m^2^ g^−1^)	V_p_ (cm^3^ g^−1^)
Bulk	10	0.48	0.063
TEX1	58	5.5	0.21
TEX2	167	15	0.66
TEX3	183	17	0.88

Note: S_m_ is the surface area of the micropores and V_p_ is the pore volume.

### Characterization by Fourier transform infrared spectroscopy

Fourier transform infrared (FTIR) spectra of bulk and exfoliated g-C_3_N_4_ are demonstrated in Figure 4. Typical vibration bands for the N-H (3300-3000 cm^−1^), C-N, and C=N (1600-800 cm^−1^) groups are prominent.^22^ The medium band at 809 cm^−1^ was attributed to the breathing mode of the triazine units, and the spectral bands around 3400 cm^−1^ were attributed to O-H stretching vibrations. Their origin can be explained by the adsorbed water and the presence of -OH groups in the g-C_3_N_4_ structures. The formation of -OH groups was also indicated by the higher oxygen and hydrogen content in the exfoliated materials (Table 1).

### Characterization by UV-Vis spectroscopy

The UV-Vis absorption spectra of the samples are shown in Figure 4. The spectra contain a single broad band peaking at ca 400 nm and are similar for both bulk and exfoliated g-C_3_N_4_. The band-gap energies were calculated according to Tauc's approach and are 2.74, 2.80, 2.80, and 2.82 eV for Bulk, TEX1, TEX2, and TEX3, respectively. For further details, see Figure S1 in the supplemental information. The slight increase in the bandgap energies (blue shift) of the exfoliated materials can be explained by the quantum confinement effect due to the decrease in the g-C_3_N_4_ particle size in terms of cracking of large g-C_3_N_4_ bulk structures into smaller nanosheets.^23^^,^^24^

### Characterization by photoluminescence spectroscopy

**Figure 5:**
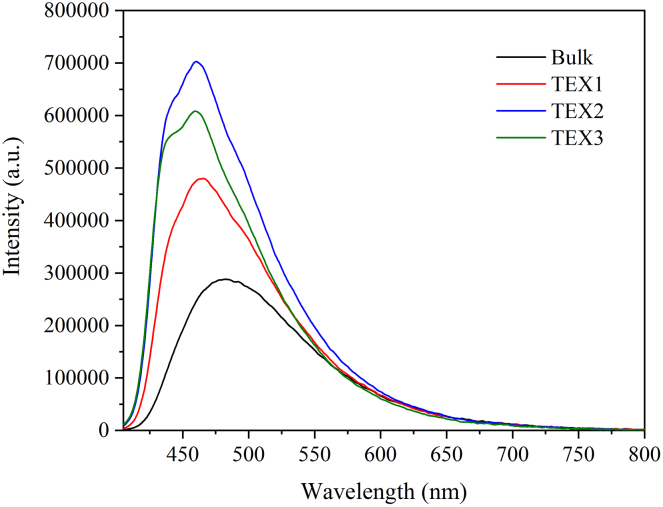
Characterization by photoluminescence spectroscopy Photoluminescence spectra of bulk and exfoliated g-C_3_N_4_ using a 450 W Xe arc lamp.

Photoluminescence (PL) spectra (Figure 5) were recorded to understand the changes in the electron-hole recombination processes within the g-C_3_N_4_ materials following thermal exfoliation. The broad PL band with a maximum at approximately 481 nm corresponds to the σ^∗^-LP and π^∗^-LP transmissions (LP denotes the nitrogen lone electron pair), and is predominantly attributed to the π–π^∗^ electron transitions observed in bulk g-C_3_N_4_.^23^^,^^25^ There is also a broad band between 500 and 550 nm, which is attributed to electron transitions between defect levels and the LP, and electron transitions between defect levels and levels related to oxygen atoms in g-C_3_N_4_, respectively.^26^

As shown in Figure 5, the maximum PL intensity first increased in the order Bulk < TEX1 < TEX2, for which the maximum PL intensity was observed and discussed later in discussion, followed by a decrease in PL intensity from TEX2 to TEX3. This can be explained by non-radiative electron transitions due to the formation of g-C_3_N_4_ structural defects. Together with the PL intensity changes, the emission bands were blue-shifted from 481 nm (Bulk) to 465 nm (TEX1) and to 461 nm (TEX2). However, the differences between TEX2 and TEX3 were minimal. The blue shift is explained in the previous section.

PL decay curves were measured to calculate the lifetimes of the photoinduced electrons and holes, and were found to follow a triple-exponential function; the results are presented in Table 4 and Figure S2. The PL lifetimes were consistent with the PL intensity, considering the first dominant PL decay component. The second “stronger” component also agrees with the PL intensity. The third component was similar for all materials and had only approximately 5–7% of the total amplitude owing to the relatively high lifetimes (∼20 ns). Therefore, the average lifetimes (τ_avg2_) were calculated for the two main components. The lifetimes of the three components (τ_avg3_) were calculated for comparison (Table 4).

**Table 4:** Fitting parameters of PL decay curves of bulk and exfoliated g-C_3_N_4_

Material	B_1_ (%)	τ_1_ (ns)	B_2_ (%)	τ_2_ (ns)	B_3_ (%)	τ_3_ (ns)	τ_avg3_ (ns)	τ_avg2_ (ns)
Bulk	61.1	1.14	33.6	4.02	5.3	19.4	8.5	3.0
TEX1	53.5	1.45	40.6	4.44	5.9	18.9	8.2	3.5
TEX2	51.9	1.74	41.4	4.95	6.7	19.8	8.8	4.0
TEX3	49.7	1.71	42.8	5.04	7.5	19.4	9.1	4.1

In general, the longer lifetimes of exfoliated g-C_3_N_4_ are indicative of more efficient separation of photoinduced electrons and holes, thereby increasing the likelihood of their participation in photocatalytic reactions. The charge separation has been confirmed by electrochemical measurements.^17^^,^^27^ The increased PL intensities and prolonged lifetimes of TEX2 and TEX3 can be attributed to the higher number of photoinduced electrons and holes, which is in agreement with the results of the photocatalytic and EPR experiments discussed later in discussion. Considering the highest PL intensities and long PL lifetimes of TEX2 and TEX3, it can be concluded that these materials are promising photocatalysts for the degradation of pharmaceuticals.

The role of vacancies in g-C_3_N_4_ is important in terms of its optical and electronic properties.^28^ The electronic properties are closely related to the photocatalytic activity. Defect engineering often leads to alterations in the conduction and valence band positions, enhancing the photocatalytic performance. Incorporating various vacancy types into g-C_3_N_4_ has been found to promote electron accumulation and facilitate surface charge transfer, creating active sites for electrochemical reactions.^29^ This defect-induced enhancement contributes to the improved photocatalytic efficiency of g-C_3_N_4_. The formation of complex vacancies in bulk and exfoliated g-C_3_N_4_ has been reported.^30^

### Reactive oxygen species and their study by electron paramagnetic resonance spectroscopy

Photocatalysis generally involves the use of a semiconductor photocatalyst that absorbs light energy and generates electron-hole pairs. Photocatalytic degradation is based on reactions involving photoinduced electrons and holes that participate in redox reactions. As photocatalytic reactions occur in the presence of oxygen and water, the photocatalytic process is accompanied by the formation of reactive oxygen species (ROS), such as the superoxide anion radical (⋅O_2_^−^), its protonated form, hydroperoxyl radical (HO_2_^⋅^), hydrogen peroxide, singlet oxygen (^1^O_2_), and hydroxyl radical (⋅OH).^31^ The main reactions leading to their formation and transformations are given later in discussion (Equations 1, 2, 3, 4, 5, and 6):

(Equation 1) $$g-C_{3}N_{4}+h\upsilon \to e^{-}\left(g-C_{3}N_{4}\right)+h^{+}\left(g-C_{3}N_{4}\right)$$

(Equation 2) $$e^{-}+O_{2}\to O_{2}^{\cdot -}$$

(Equation 3) $$O_{2}^{\cdot -}+H^{+}⇆{HO}_{2}^{\cdot }\;\left(\text{pK}=4.8\right)$$

(Equation 4) $$2\;{HO}_{2}^{\cdot }\to H_{2}O_{2}+O_{2}$$

(Equation 5) $$H_{2}O_{2}+e^{-}\to {\text{OH}}^{\cdot }+{\text{OH}}^{-}$$

(Equation 6) $$H_{2}O_{2}+hv\to {2\;\text{OH}}^{\cdot }$$

In addition to ROS, holes can also react with organic substances as oxidising species^32^^,^^33^; therefore, the overall photocatalytic process can be written as follows:

(Equation 7) $$\text{ROS}+h^{+}+\text{organic}\;\text{substances}\to \text{degradation}\;\text{products}+{\text{CO}}_{2}+H_{2}O$$

The important role of hydrogen peroxide in the photocatalytic degradation of organic compounds has been discussed in the literature.^34^^,^^35^ As hydrogen peroxide is a stable substance, the stability of g-C_3_N_4_ was tested by treating TEX2 with 10–30% H_2_O_2_ solutions for 5 h, followed by structural (FTIR, XRD, and XPS) analyses, as shown in Figures S3–S5. No g-C_3_N_4_ instability was observed. This is in line with the findings of many studies, which have reported the photocatalytic stability of g-C_3_N_4_ used for the degradation of organic substances, for example.^36^

The generation of superoxide and hydroxyl radicals upon the irradiation of the g-C_3_N_4_ samples was studied by spin trapping using 3,4-dihydro-2,3-dimethyl-2H-pyrrole 1-oxide (DMPO). DMPO forms a stable spin adduct with hydroxyl radicals (DMPO-OH) featuring a four-line electron paramagnetic resonance (EPR) spectrum with 1:2:2:1 line intensities. The DMPO adduct with superoxide radical anion (DMPO–OOH) features a 12-line spectrum^37^ that is usually not fully resolved owing to broadening effects, merging the features into sextets or quartets. Moreover, the adduct is unstable and may transform into DMPO–OH after some time period. Therefore, EPR experiments were performed in a mixed DMSO/water solvent, where the stability of the primary photogenerated superoxide radical anion is increased, as reported in.^38^ EPR spectra were acquired consecutively at 30 s intervals for bulk g-C_3_N_4_ and exfoliated samples TEX1-3 under constant intensity irradiation (Figures S6–S9). The control experiment was performed by irradiating the DMPO solution in the absence of g-C_3_N_4_ (Figure S9).

**Figure 6:**
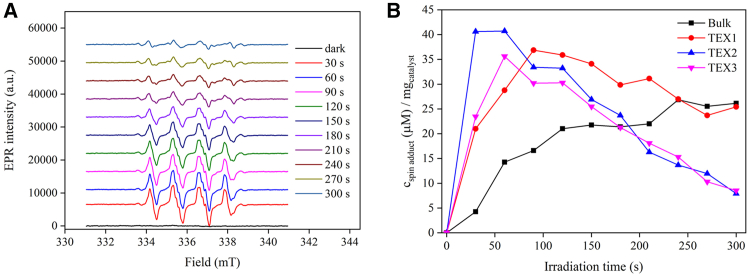
Characterization by EPR spectroscopy (A) EPR spectra obtained from the DMPO spin trapping experiment with TEX2 g-C_3_N_4_. (B) The time development of the normalized EPR signal induced by the irradiation of bulk and exfoliated g-C_3_N_4_. The acquisition time of each spectrum was 30 s with a modulation amplitude of 0.2 mT and a microwave attenuation factor of 10 dB.

The results show that in the absence of g-C_3_N_4_, no spin adducts were formed. In contrast, both bulk and exfoliated g-C_3_N_4_ samples produced the same primary radical or a mixture of primary radicals, as indicated by the identical EPR spectra of the DMPO spin adducts, differing only in intensity. The kinetics of spin adduct formation were markedly different between the bulk and exfoliated samples (Figure 6). At a given irradiation intensity, the initial rates of spin adduct formation were determined to be 0.14, 0.70, 1.35, and 0.78 μmol L^−1^ s^−1^·mg^−1^ for the Bulk, TEX1, TEX2, and TEX3 samples, respectively.

In the 5-min EPR experiment, a gradual build-up of the EPR signal was observed, while exfoliated g-C_3_N_4_ provided a sharp increase in the EPR intensity followed by decay, indicating the photocatalytic decomposition of the DMPO spin adduct present in the EPR capillary. The fastest build-up and decay of the EPR signal was observed for TEX2 (maximum EPR intensity was observed at 30 s), while TEX1 provides maximum at 90 s and TEX3 at 60 s. It can be concluded that superoxide anion radicals were the primary reactive oxygen species produced by both bulk and exfoliated g-C_3_N_4_ samples. The EPR experiments also indicated that TEX2 was the most efficient photocatalyst.

### Photocatalytic degradation of pharmaceuticals

**Figure 7:**
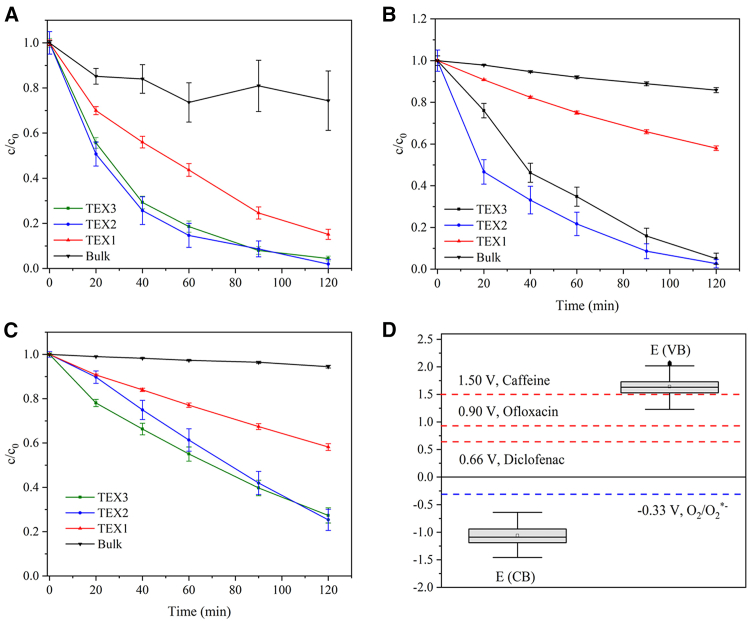
Photocatalytic degradation of pharmaceuticals Kinetic curves of the photocatalytic degradation of (A) ofloxacin, (B) diclofenac, and (C) caffeine using Bulk, TEX1, TEX2, and TEX3 photocatalysts and (D) energy diagram. An LED source of 420 nm and an intensity of 13.5 mW·cm^−2^ was used.

The bulk and exfoliated g-C_3_N_4_ samples were used as photocatalysts for the degradation of the model pharmaceuticals ofloxacin, diclofenac, and caffeine. In the dark, suspensions of g-C_3_N_4_ and the pharmaceutical were first stirred for 60 min to reach adsorption-desorption equilibrium. The suspensions were then stirred under 420 nm irradiation for 120 min. The kinetic curves are presented in Figure 7.

The selected pharmaceuticals were phototactically degraded by their oxidation according to the reaction (7). The degradation of all pharmaceuticals followed first-order kinetics:

(Equation 8) $$-\frac{dc}{dt}=kc$$

and

(Equation 9) $$c=c_{0}\;\exp\left(-kt\right)$$

where *k* is the rate constant, and *c* and *c*_*0*_ are the concentrations of pharmaceuticals at time *t* = *t* and *t* = 0, respectively. The rate constants are listed in Table 5. Their values increased in the sequence Bulk < TEX1 < TEX3 < TEX2. The degradation efficiency decreased in the following order: ofloxacin > diclofenac > caffeine. After 120 min, more than 95% of ofloxacin and diclofenac and approximately 80% of caffeine were decomposed.

**Table 5:** Rate constants (k) of photocatalytic degradation of pharmaceuticals

Substance	Bulk k × 10^−3^ (min^−1^)	TEX1 k × 10^−3^ (min^−1^)	TEX2 k × 10^−3^ (min^−1^)	TEX3 k × 10^−3^ (min^−1^)
Ofloxacin	4.67 ± 0.96	15.5 ± 0.6	27.5 ± 2.3	26.2 ± 1.0
Diclofenac	1.30 ± 0.06	4.54 ± 0.08	28.7 ± 2.6	20.4 ± 1.3
Caffeine	0.453 ± 0.027	4.45 ± 0.11	12.6 ± 0.9	10.5 ± 0.5

The photocatalytic results were compared with those reported in the literature for the different photocatalysts listed in Table S3. The experimental conditions under which the reported results were obtained varied in terms of reactor geometry, irradiation intensity and energy, reaction time, pharmaceutical concentration, photocatalyst mass, and photocatalyst composition. Therefore, it is impossible to compare them accurately; however, these studies suggest that simple thermally exfoliated g-C_3_N_4_ provides comparable photocatalytic results.

Photocatalytic processes usually obey the Langmuir-Hinshelwood mechanism, which assumes that two molecules adsorb on neighboring sites of a photocatalyst and then react with each other. Thermal exfoliation increased the specific surface area of g-C_3_N_4_ from 10 to 184 m^2^g^-1^ (Table 3), leading to the formation of a higher number of reaction sites on the material surface and an increase in the electron–hole lifetime (Table 4). In addition, the PL intensity indicates a higher number of electrons and holes produced by the exfoliated g-C_3_N_4_. The number of reaction sites, charge carriers, and their lifetimes are important parameters for suitable photocatalysts and, in turn, for efficient photocatalytic degradation. A comparison of all three parameters (Figure S10), TEX2 was identified as the optimum photocatalyst.

The different degradation rates of individual pharmaceuticals can be discussed in terms of their electrochemical redox potentials. The values of 0.90 V for ofloxacin,^39^ 0.66 V for diclofenac,^40^ and 1.50 V for caffeine^41^ (measured at pH 7 vs. the Ag/AgCl reference electrode) are available in the literature. Recently, graphitic carbon nitride was investigated to have the valence band potentials (E_VB_) of 1.54–1.65 V and the conduction band potential (E_CB_) from −1.10 to −1.19 V^42^; therefore, photoinduced electrons are able to react with oxygen dissolved in water, resulting in the formation of superoxide radicals with a redox potential of −0.33 V.^43^ These potentials agree well with the calculated medians of E_VB_ and E_CB_ from the literature data of 1.63 V and −1.09 V (*n* = 98), respectively,^44^ which are displayed in Figure 7.

**Figure 8:**
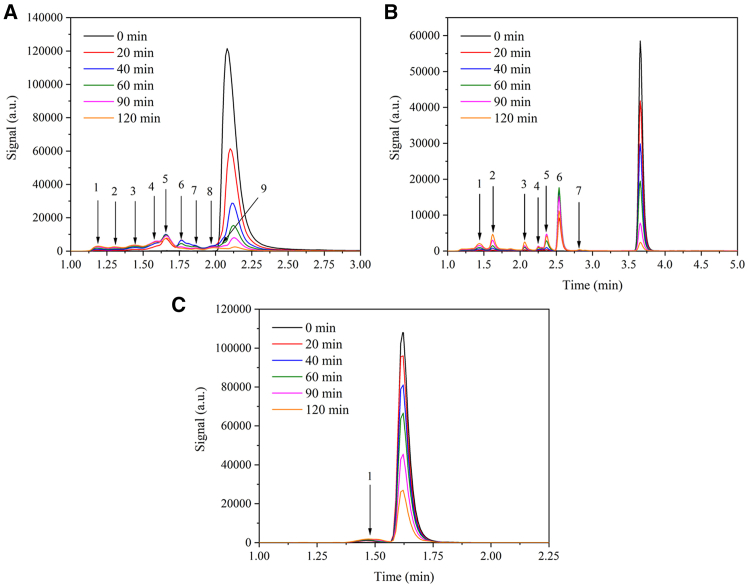
Analysis of degradation products HPLC chromatograms of (A) ofloxacin, (B) diclofenac, and (C) caffeine during the photocatalytic degradation using TEX2 at 420 nm. The column of 150 mm length, 4.6 mm i.d. (2.6 μm core-shell particles) and the mobile phase of 0.1% formic acid in water and a mixture of acetonitrile and methanol (85:15, v/v) in a ratio of 40:60 (v/v) were used.

The thermodynamic data indicate that oxidation by reactive oxygen species (ROS) is feasible for all pharmaceuticals. Similarly, the photoinduced holes oxidized ofloxacin and diclofenac. However, the involvement of holes in the oxidation of caffeine is likely limited because the oxidation potential of caffeine (E^o^ = 1.50 V) and the E_VB_ values of g-C_3_N_4_ (E_VB_ = 1.63 V) are similar. Furthermore, the lower degradation of caffeine can also be explained by the competitive adsorption/degradation behavior between caffeine molecules and their degradation intermediates.^45^^,^^46^

Ofloxacin degraded better than diclofenac, even though its redox potential is higher. This can be explained by the adsorption of these pharmaceuticals onto the photocatalysts. As shown in Figure S11, ofloxacin was adsorbed more than diclofenac and caffeine, which were weakly adsorbed. In general, surface catalytic reactions are based on the adsorption of reactants; therefore, the adsorption of the studied pharmaceuticals is equally important for their photocatalytic degradation as their redox potentials. The stability of g-C_3_N_4_ during the photocatalytic reactions was confirmed by XRD and XPS analyses. The similarity of the XRD patterns and XPS spectra of the exfoliated g-C_3_N_4_ before and after the degradation of ofloxacin (Figures S12 and S13) confirms the photocatalyst stability and is consistent with the stability against hydrogen peroxide discussed sooner.

### Products of photocatalytic degradation

The determination of intermediate degradation products is a challenging issue in photocatalytic investigations. The reaction mixtures were analyzed during the photocatalytic experiment using high-performance liquid chromatography (HPLC) equipped with a photodiode array (PDA) detector. Figure 8 shows the HPLC chromatograms of the photocatalytic degradation over time.

The decrease in the main peaks of the tested pharmaceuticals is evident. Moreover, other minor peaks of the degradation products (labeled by numbers in Figure 8) were observed which eluted at shorter retention times. Low levels of these signals imply that the pharmaceuticals were degraded.

Unlike caffeine, where only a small amount of intermediary degradation products manifested as a single broad HPLC peak, the chromatograms of diclofenac contained seven intermediates, whereas in the case of ofloxacin, nine peaks were observed. Over the course of the degradation, some of these peaks increased and some decreased in height. As the degradation products are continually transformed from one to another, they are hereafter denoted as transformation products (TPs). In general, all the TPs of all the studied pharmaceuticals remained in the chromatograms of the respective reaction mixtures irradiated for 120 min. This allowed the HPLC combined with high-resolution tandem mass spectrometry (HPLC/HRTMS) identification of individual pharmaceutical TPs from a single reaction mixture.

### Transformation pathway of degradation products

**Figure 9:**
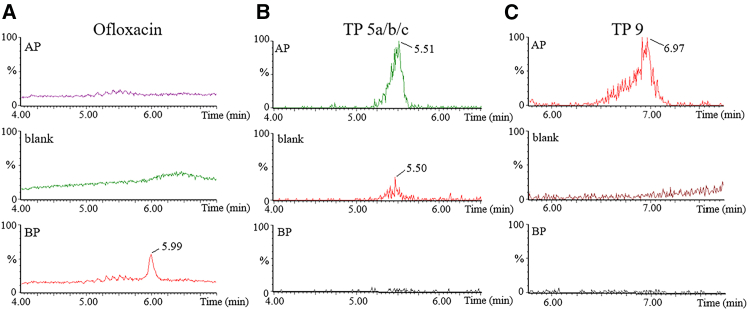
Identification of the transformation pathways of degradation products. HPLC/HRTMS chromatograms of ofloxacin (A) Top: ofloxacin in AP sample, middle: blank sample, bottom: ofloxacin in BP sample. (B) Top: TP 5a/b/c in AP sample, middle: TP 5a/b/c in blank sample, bottom: TP 5 a/b/c in BP sample. (C) Top: TP 9 in AP sample; middle: TP 9 in blank sample, bottom: TP 9 in BP sample. The column of 150 mm length, 4.6 mm i.d. (2.6 μm core-shell particles) and the mobile phase of 0.1% formic acid in water and a mixture of acetonitrile and methanol (85:15, v/v) in a ratio of 40:60 (v/v) were used.

For each pharmaceutical, aliquots were taken from the reaction mixtures after 120 min of photocatalysis (AP) and analyzed by HPLC/HRTMS in full scan mode. For control, the original reaction mixture before photocatalysis (BP), blank, and standard samples (for a detailed description of the samples, refer to the Experimental section) were also measured. The representative chromatograms of ofloxacin are shown in Figure 9.

Significant signals that were present only in the AP solution (compared to the standard, blank, and BP solutions) were considered to be significant. The detected signals (m/z) were then fragmented, and the rising fragments in the high-resolution MS/MS spectra were investigated to determine the chemical structures of the TPs. The final TP molecules were identified, and the mass errors were calculated. Finally, possible transformation mechanisms were proposed to elucidate the photocatalytic transformation processes of each pharmaceutical.

#### Transformation pathway of ofloxacin

**Scheme 1:**
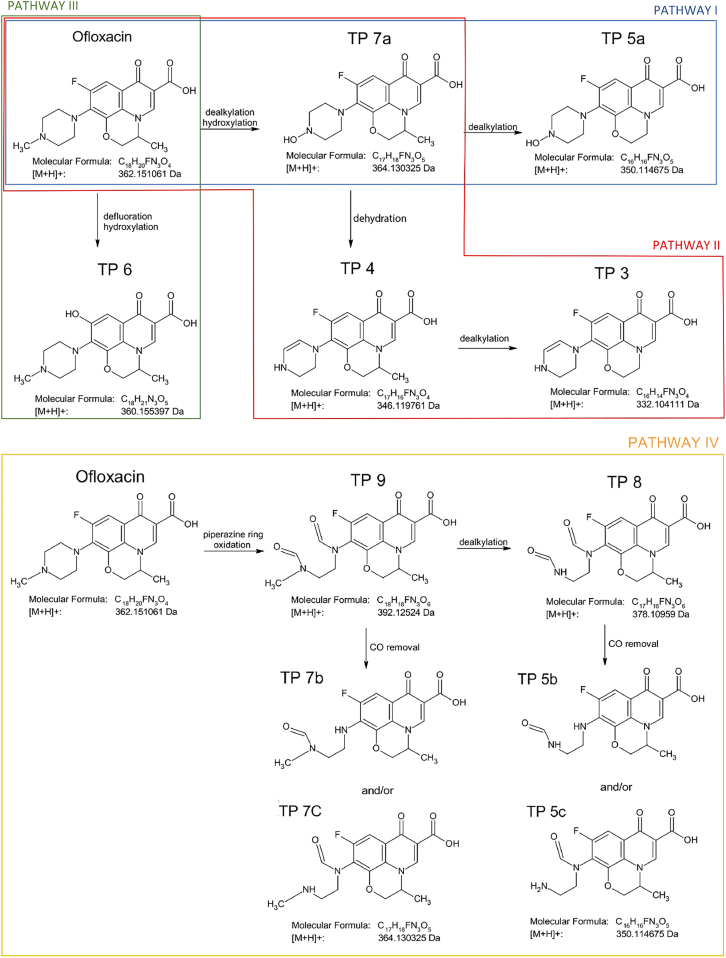
Proposed transformation pathways for the photocatalytic degradation of ofloxacin

Thirteen TPs were identified for ofloxacin (Table S4). Three structures corresponding to *m/z* 364.1278 and three structures corresponding to m/z 350.1143 are labeled with numbers 7 and 5, to which small letters (a, b, c) are added, respectively. We were able to propose four transformation pathways, which included 11 out of 13 identified TPs (TP3-TP9, see Scheme 1); TP 1 and TP 2 did not directly fit any of the pathways, so they are listed separately (Table S4). During the photocatalytic degradation, ofloxacin lost the carboxyl group, and the methylpiperazine ring was cleaved to varying degrees without further oxidation, resulting in the formation of TP 1 and TP 2. The mechanism leading to these TPs appears to be fragmentation rather than oxidation, and these TPs have not been previously documented in the literature.

The TPs appear to follow the traditional principles of ROS-mediated reactions. In other words, hydroxylation, oxidation, and dealkylation caused by the attack of ROS are the most commonly observed processes in the degradation of ofloxacin, which is in agreement with previously published works.^47^^,^^48^^,^^49^^,^^50^^,^^51^^,^^52^ According to the Fukui function, which is based on density functional theory (DFT) calculations and is used to predict the sites most prone to radical reactions, the most likely site to be attacked is a methylpiperazine ring, more specifically, two nitrogen atoms,^47^^,^^49^ which also corresponds with our findings. Three of the four proposed transformation pathways are based on the oxidation or destruction of the original piperazine ring structure.

In pathway I, a hydroxyl group substitutes the methyl group on the piperazine ring, leading to TP 7a. Subsequently, another dealkylation step occurred on the morpholine ring, resulting in TP 5a. Similar mechanisms have been reported in the literature.^48^^,^^50^^,^^51^^,^^52^ Pathway II leads to the same TP 7a as pathway I; however, before the dealkylation of the morpholine ring (TP 3), dehydration of the piperazine ring occurs, resulting in double bond formation (TP 4). Pathway III is the only process in which one can see a cleavage of a carbon-fluorine bond, with subsequent hydroxylation of the same carbon, resulting in the formation of TP 6. Despite the fact that the C-F bond is one of the strongest bonds in organic chemistry, the phenomenon of fluorine cleavage has been previously reported in the relevant literature (and furthermore supported by the strong fluorine ion yield observed during the photocatalysis).^49^

The final pathway, IV, is initiated by the cleavage and oxidation of the piperazine ring (TP 9). Then, CO is eliminated from one carbon atom or another, with the subsequent formation of TP 7b and/or TP 7c. TP 5b and/or TP 5c were formed using a similar process. Dealkylation then occurs on the oxidized ring, leading to TP 8. Thus, transformation processes similar to those proposed in this study have been observed previously, even when using other photocatalysts such as TiO_2_ and g-C_3_N_4_ heterocomposites.^47^^,^^48^^,^^49^^,^^50^^,^^51^^,^^52^ This suggests that common degradation mechanisms are mediated by ROS and h^+^ during ofloxacin removal. We were able to identify only some parts of the degradation process. Presumably, later in the process, molecules with lower molecular masses were formed (we were able to identify two such molecules, TP 1 and TP 2), and the entire process ended with the total oxidation of ofloxacin to CO_2_ and H_2_O.

**Figure 10:**
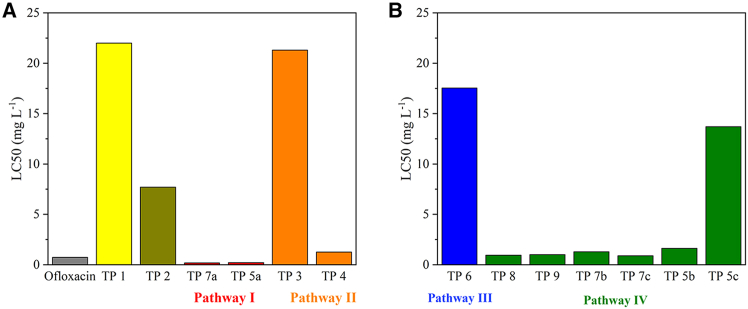
QSAR-predicted ecotoxicity of ofloxacin and its transformation products (A) Ofloxacin, TP 1, TP 2, TP 7a, TP 5a, TP 3, and TP 4. (B) TP 6, TP 8, TP 9, TP 7b, TP 7c, TP 5b, and TP 5c. LC50 for *Pimephales promelas*, 96 h. Most TPs show lower toxicity than the parent compound, indicating that three of the four degradation pathways can be considered environmentally safe.

In general, the degradation of pharmaceuticals, which can be harmful and toxic to the environment, is the goal of photocatalytic experiments; however, the toxicity of the transformation products should also be considered. Quantitative structure–activity relationship (QSAR) models have been widely used to predict the potential toxicity of molecules. Although such *in silico* approaches do not fully replace *in vivo* methods, which are often costly and time-consuming, they can provide reliable ecotoxicological assessments. The acute toxicity (LC50, 96h) of all identified TPs (including TP 1 and TP 2, which were not included in the tentative degradation pathways) and the original molecules was investigated for the model organism fathead minnow (*Pimephales promelas*), and the results are reported in Figure 10.

The higher the LC50, the lower the acute toxicity. In the case of ofloxacin and its transformation products, only TP 7a and TP 5a had lower LC50 values and were therefore more toxic; the remaining TPs were less harmful to the aquatic environment than ofloxacin, proving that three of the four degradation pathways can be considered environmentally safe. Similar results for the TPs identified in this study have been reported in the literature.^47^^,^^49^^,^^51^

#### Transformation pathway of caffeine

**Scheme 2:**
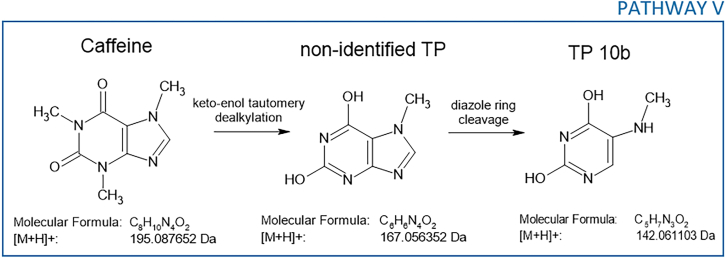
Proposed transformation pathway for the photocatalytic degradation of caffeine

The HPLC/PDA chromatograms depicting caffeine degradation revealed the presence of only one additional peak (Figure 8). The identification of a similar number of TPs and only one m/z value, which could correspond to either TP 10a or TP 10b, was expected. TP 10a, whose structure and complementary data are shown in Table S5, was obtained by demethylation and cleavage of the diazole ring of caffeine. TP 10b, with the same *m/z* 142.0642 as TP 10a, results from the degradation of the keto-enol tautomeric form of caffeine that underwent demethylation on both pyrimidine ring nitrogen atoms. In similar studies dealing with the degradation of caffeine, no match with these TPs was found, and surprisingly, TP 10a and/or b did not fit well into the predicted transformation pathways, as they did not show any signs of further oxidation.^53^^,^^54^^,^^55^^,^^56^ One of the most commonly identified TP in the photocatalytic degradation of caffeine is dimethylparabanic acid, with a theoretical *m/z* of 143.0451^57^; however, this molecule was not detected in our study. Only one transformation pathway, V, described in Scheme 2, was proposed, which only involved TP 10b and its undetected precursor.

**Figure 11:**
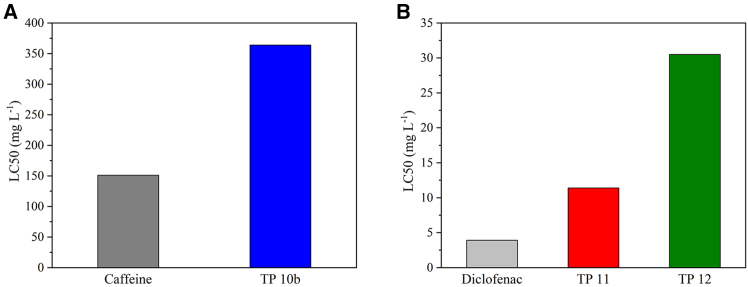
QSAR-predicted ecotoxicity of caffeine, diclofenac, and their transformation products (A) Caffeine, TP 10b. (B) Diclofenac, TP11, and TP 12. LC50 for *Pimephales promelas*, 96 h. TP 10b, TP 11, and TP 12 exhibit higher LC50 values than their parent compounds, indicating reduced acute toxicity. Photocatalytic degradation thus decreases the ecotoxicity and improves the environmental safety of the resulting TPs.

Based on the literature, degradation is expected to result in the terminal products of CO_2_, H_2_O, and NH_3_.^53^ The toxicity of caffeine and its TPs differs among species. In humans, the acute and chronic toxicity of caffeine is quite low, as proven by the daily intake of caffeine in coffee, tea, and other beverages, which has no serious consequences for humans. For rats, caffeine is also considered, not harmful” with the LD50 of 190 mg L^−1^, on the other hand, it was labeled as, very toxic” to green algae.^57^ According to QSAR, the LC50 of TP 10b for fathead minnows is higher than that LC50 of caffeine (151 mg·L^−1^ and 364 mg·L^−1^, respectively; see Figure 11); thus, the low ecotoxicity of caffeine for these organisms was further reduced by photocatalysis.

#### Transformation pathway of diclofenac

**Scheme 3:**
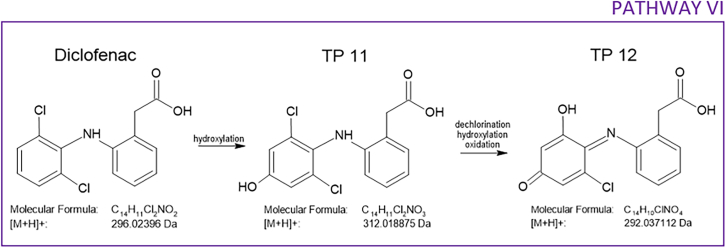
Proposed transformation pathway for the photocatalytic degradation of diclofenac

The photocatalytic degradation of diclofenac is associated with the formation of hydroxylated and further oxidized TPs. We identified two TPs, both of which have been previously described^50^^,^^58^ (Table S6). The proposed transformation pathway VI, shown in Scheme 3, describes the oxidation processes that occur on a dechlorinated aromatic ring. First, TP 11 is created by the hydroxylation of a carbon atom, which, according to the Fukui function calculations, is most prone to ROS attack.^58^ According to similar studies, hydroxylation can also occur on other carbon atoms.^48^^,^^49^ Furthermore, one study suggested that three different hydroxylated products can be formed depending on the hydroxyl group placement.^50^ TP 12 is formed upon dechlorination and subsequent hydroxylation of chlorine-free carbon, followed by the oxidation of the previously attached hydroxyl group.^51^^,^^58^ No additional TPs were identified. However, according to the literature, a deeper degradation process continues with further oxidation, eventually resulting in the formation of carboxylic acids and the final mineralization to CO_2_ and H_2_O.^48^

QSAR predictions revealed the reduced aquatic ecotoxicity of both TPs compared to ofloxacin; the LC50 of TP 11 and TP 12 was higher than that LC50 of the original substance (Figure 11). These findings have also been reported previously in the literature; furthermore, the photocatalytic degradation decreased the bioaccumulation factor and mutagenicity of the TPs.^48^^,^^58^ In other words, both identified TPs are safer for the environment than diclofenac.

### Conclusion

Bulk graphitic carbon nitride was synthesized via dicyandiamide thermolysis at 550 ^°^C. Exfoliated materials were prepared by the further calcination of bulk g-C_3_N_4_ at 500 °C for 1–3 h (TEX1-3). The prepared materials were characterized by elemental analysis, SEM and TEM imaging, XRD, physisorption of nitrogen, XPS, FTIR, UV-Vis, photoluminescence, and EPR spectroscopy.

The exfoliation of g-C_3_N_4_ provided a higher specific surface area, leading to more reaction sites and longer lifetimes of the photoinduced electrons and holes for the photocatalytic reactions. However, excessive thermal exfoliation led to the degradation of graphitic carbon nitride by the formation of more defects (TEX3), as documented by structural, textural, and spectroscopic analyses. Under the specific conditions of this study, the optimum exfoliation time was determined to be 2 h. The corresponding material provided a relatively high S_BET_, lifetime, and the highest PL intensity, which was related to the highest number of electrons and holes. It was the most active photocatalyst in this study, as confirmed by the photocatalytic degradation of ofloxacin, diclofenac, and caffeine under irradiation at 420 nm.

The superoxide anion radical was identified as the primary reactive oxygen species mediating degradation using EPR spectroscopy. The degradation rate decreased in the order of ofloxacin > diclofenac > caffeine. After 120 min, more than 95% of ofloxacin and diclofenac and approximately 80% of caffeine were degraded. Although most of the pharmaceuticals were removed, photocatalytic investigations were further focused on identifying intermediary degradation products using HPLC/HRTMS. Based on the identified intermediates, transformation pathways were proposed.

This study confirms that exfoliated g-C_3_N_4_ is a suitable metal-free, low-cost, and easy-to-prepare photocatalyst that can be activated by visible light and has potential in green technology water treatment. This also demonstrates that not only the degradation rate of the original organic compounds but also their degradation products need to be investigated. Despite significant progress, a knowledge gap remains regarding the degradation products generated by photocatalysis and, more broadly, advanced oxidation processes (AOPs). Sensitive analytical techniques are essential for the accurate identification and quantification of these transformation products.

### Limitations of the study

The degradation products in this study were identified using HPLC/PDA and HPLC/HRTMS. While effective for the analysis of various intermediates, these analytical methods are limited by their detection and quantification limits, which may prevent the identification of trace-level transformation products. Furthermore, carbon dioxide, which is reported to be the final mineralization product of organic compounds such as pharmaceuticals, could not be identified in the photocatalytic setup. Further experiments with specially designed equipment should be performed.

Another limitation is the number of tested pharmaceuticals. This study was focused on three representative compounds, namely, ofloxacin, diclofenac, and caffeine, which were chosen because of their frequent occurrence in wastewater. Broader studies, including other antibiotics, hormones, and psychiatric pharmaceuticals, would provide deeper insights into the photocatalytic degradation of pharmaceuticals and their transformation pathways.

## Resource availability

### Lead contact

Requests for further information and resources should be directed to and will be fulfilled by the lead contact, Petr Praus (petr.praus@vsb.cz).

### Materials availability

This study did not generate new unique reagents.

### Data and code availability

This article does not report original code. All data associated with this study are present in the article or the supplemental information. Any additional information required to reanalyze the data reported in this article is available from the lead contact upon request.

## Acknowledgments

This study was financially supported by the 10.13039/501100000780European Union under the REFRESH – Research Excellence For REgion Sustainability and High-tech Industries (Project No. CZ.10.03.01/00/22_003/0000048) via the Operational Programme Just Transition and by the OP JAK project “INOVO!!!” (No. CZ.02.01.01/00/23_021/0008588) provided by the Ministry of Education, Youth, and Sports (Czech Republic) and co-financed by the 10.13039/501100000780European Union. The authors also thank the Large Research Infrastructure ENREGAT (Project No. LM2023056). The authors wish to thank Prof. Dr. J. Vinklárek from the 10.13039/501100016365University of Pardubice (Czech Republic) for providing access to the EPR facility and Dr. M. Koštejn from the Institute of Chemical Process Fundamentals (Czech Academy of Science) for providing XPS spectra.

## Author contributions

P.P. performed conceptualization, supervision, data curation, investigation, formal analysis, and article writing. A.G., J.H., and P.B. performed the investigation, data curation, and article writing. K.S. performed investigation and data curation.

## Declaration of interests

The authors declare no competing interests.

## Star★Methods

### Key resources table

**Table:** 

REAGENT or RESOURCE	SOURCE	IDENTIFIER
**Chemicals, peptides, and recombinant proteins**		

Ofloxacin	Merck	33703
Diclofenac	Merck	SML3086
Caffeine	Merck	C0100000
Dicyandiamide (DCDA)	Merck	D76609
Acetonitrile	Supelco	1.00030
Formic acid	Supelco	1.00029
4-hydroxy-2,2,6,6-tetramethyl-1-piperidinyloxyl (TEMPOL)	Aldrich	S332216
Dimethyl sulfoxide (DMSO)	Merck	472301
5,5-dimethyl-1-pyrroline N-oxide (DMPO)	Merck	92688
Methanol	Merck	646377

**Software and algorithms**		

QSAR Toolbox	OECD and ECHA	4.6

### Experimental model and study participant details

Most of the experiments and tests were performed at VSB-Technical University of Ostrava. EPR and XPS experiments were performed at University of Pardubice and the Czech Academy of Science, respectively. All the experiments involve no laboratory animals.

### Method details

#### Synthesis of g-C_3_N_4_

Bulk g-C_3_N_4_ was synthesized by direct heating of 5 g of DCDA placed in a ceramic crucible with a lid in a muffle furnace and heated at a rate of 3 °C·min^−1^ from room temperature to 550 °C, the total heating time was 4 h. The crucible was then transferred out of the furnace and cooled to room temperature. The as-prepared bulk g-C_3_N_4_ was ground into a fine powder using a laboratory mill. Bulk g-C_3_N_4_ was exfoliated by heating a thin layer spread on a ceramic plate (diameter 8 cm, 50 mL) in a muffle furnace for 1–3 h (initial heating rate of 10 °C·min^−1^, final temperature 500 °C). The ceramic plate containing the product was then cooled to ambient temperature. The exfoliated materials were labeled TEX1, TEX2, and TEX3 according to the exfoliation time (1, 2, and 3 h).

The stability of the TEX photocatalysts toward hydrogen peroxide was tested using TEX2 and 10–30% H_2_O_2_ solutions. One gram of TEX2 was placed in a 100 mL glass autoclave together with 50 mL of H_2_O_2_ solution and stirred for 5 min. After stirring, the autoclave was placed in an oven at 150 °C for 5 h. The resulting TEX2 was filtered, washed several times with deionised water, and dried overnight at 105 °C.

The stability of TEX3 during the photocatalytic process was tested over 3 cycles of the photocatalytic degradation of ofloxacin. The material was filtered after every cycle (120 min), washed with distilled water, and dried at 105°C in a laboratory drier.

#### Elemental analysis

The C, H, and N contents in the g-C_3_N_4_ materials were determined using a Flash 2000 elemental analyser (ThermoFisher Scientific, Waltham, MA, USA). The oxygen content was estimated as the remaining fraction after subtracting the measured C, H, and N content from 100%.

#### Scanning and transmission electron microscopy

Scanning electron microscopy and energy-dispersive X-ray spectroscopy (EDS) analyses of the g-C_3_N_4_ materials were performed using a Tescan Vega microscope (Brno, Czech Republic) equipped with a tungsten cathode. SEM micrographs were obtained using secondary electron (SE) and backscattered electron (BSE) modes with an acceleration voltage of 30 keV. The samples were gold-sputtered before analysis to ensure adequate electron conductivity.

Transmission electron microscopy was performed using a JEOL 2100 microscope equipped with a LaB_6_ electron gun. An accelerating voltage of 200 kV was applied. TEM micrographs were recorded using a Tengra camera (EMSIS). For the TEM analysis, the samples were dispersed in ethanol and sonicated for 5 min. One drop of this solution was placed on a copper grid with a holey carbon film and dried at room temperature.

#### X-Ray diffraction analysis

X-ray diffraction patterns were recorded using a Rigaku SmartLab diffractometer (Rigaku, Tokyo, Japan) equipped with a D/tex Ultra 250 detector. The X-ray irradiation source was a Cu tube (CuK_α_, λ_1_ = 0.154059 nm, λ_2_ = 0.154441 nm) operated at 40 kV and 30 mA. The incident and diffracted beam optics were equipped with 5 deg Soller slits, and the incident slits were set to 1 mm. The powder samples were gently ground using an agate mortar, pressed by a microscope glass in a rotational sample holder, and measured in reflection mode (Bragg-Brentano geometry). The samples were rotated (30 rpm) to eliminate the effects of the preferred orientation. The XRD patterns were collected in the 2θ range of 5–90 deg with a step size of 0.01 deg and a speed of 0.5 min^−1^. Measured XRD patterns were evaluated using PDXL 2 software (version 2.4.2.0, Rigaku, Tokyo, Japan) and compared with a database PDF-2, release 2015 (ICDD, Newton Square, USA). A crystallite size L was obtained from Scherrer's equation

$$B\left(2\theta \right)=\frac{K\lambda }{Lcos\theta }$$

where B(2θ) is a broadening B(2θ) (in radians) at a half-maximum intensity (FWHM) of a diffraction band, λ is the wavelength of X-rays, θ is Bragg's angle, and K is a constant equal to 0.94 for cubic or 0.89 for spherical crystallites (the value K = 0.90 was used in this work).

#### X-ray photoelectron spectroscopy

X-ray photoelectron spectroscopy was performed using a spectrometer ESCA 3400 (Kratos Analytical Ltd, UK) with a base pressure in an analysis chamber of 5.0 × 10^−7^ Pa. The powdered materials were placed on conductive carbon tape and analyzed. The electrons were excited using Mg K_α_ radiation (hν = 1253.6 eV) generated at 12 kV and 10 mA, respectively. For all spectra, the Shirley background was subtracted. Peaks ascribed to sp^2^ hybridised nitrogen (C=N-C) were set to 398.8 eV for charge correction.

#### Physisorption of nitrogen

The specific surface area was measured by physisorption of nitrogen at 77 K using a 3Flex apparatus (Micromeritics, USA). Before the analysis, each material was degassed under vacuum (approximately 0.6 bar at 105 °C for 120 h (5 days)) to release physisorbed water and organic residuals from the pores. After this pre-treatment, the nitrogen adsorption-desorption isotherms of all materials were measured at 77 K in a relative pressure range of p/p_0_ ∼10^−9^-0.99. The nitrogen adsorption–desorption data were treated according to the classical BET theory (for p/p_0_ ≈ 0.05–0.25). The Barrett-Joiner-Halenda (BJH) model was used to calculate the pore size distributions of the materials. The Carbon Black STSA standard isotherm (typically used for carbon-based materials) was used for comparison using the Faas correction and assuming a cylindrical-pore geometry of mesopores and macropores.

#### Fourier transform infrared spectroscopy

Fourier transform infrared spectroscopy was performed using a Nicolet iS50 device (Thermo Scientific, Waltham, MA, USA) with the KBr pellet technique. The powder sample was mixed and homogenised with KBr (approximately 200 mg) and pressed at a pressure of 20 MPa to obtain a transmission pellet. The prepared pellet was placed in the holder of a transmission attachment, and the FTIR spectra were collected in the wavenumber range of 500–4000 cm^−1^ with a resolution of 2 cm^−1^. Each spectrum consisted of at least 64 scans, each lasting 1 s. Before each measurement, the background was collected to eliminate the effects of the apparatus and environment.

#### UV-Vis spectroscopy

UV-Vis diffuse reflectance spectroscopy was performed in the range of 300–1000 nm using a Specord 250 (Analytik Jena, Germany) instrument equipped with an integrating sphere (Analytik Jena, type 820-60139-P). The absorbance data were evaluated using Aspect UV software. Band gap energies were calculated using from Tauc’s plots as

$$Ah\nu ={C\left(h\nu -E_{g}\right)}^{p}$$

where *A* is the absorbance, *hν* is the energy of the incident photons, *E*_*g*_ is the bandgap energy, *C* is a constant, and *p* is a power quotient depending on the type of electron transition (p = ½ was used in this study).

#### Photoluminescence spectroscopy

Photoluminescence spectroscopy measurements were performed on an FLS980 fluorescence spectrometer (Edinburgh Instruments) with double monochromators on both the excitation and emission sides, equipped with an R928P photomultiplier in a thermoelectrically cooled housing (Hamamatsu Photonics). A 450 W xenon arc lamp served as the excitation source for the steady-state spectra. Spectral correction curves were obtained from the Edinburgh Instruments. The powder samples were mounted onto a front-face quartz sample holder.

For time-resolved measurements, an EPL-375 ps pulsed diode laser (λ_em_ = 372 nm) with a pulse width of 66.5 ps, repetition rate of 20 MHz, and average power of 75 μW (Edinburgh Instruments) was used in conjunction with a time-correlated single-photon counting (TCSPC) system. Obtained PL decay curves were fitted using a triple exponential function

$$I\left(t\right)=\sum_{i=1}^{3}B_{i}\;\exp\left(-\frac{t}{\tau_{i}}\right),where\;\sum_{i=1}^{3}B_{i}=1$$

In this expression, *τ*_*i*_ represents the decay time constant, and *B*_*i*_ represents the normalised amplitude of each component. The amplitude-weighted average decay lifetime *τ*_*avg*_ of the entire fluorescence decay process was calculated as follows:

$$\tau_{avg}=\frac{\tau_{i}^{2}B_{i}}{\sum \tau_{i}B_{i}}$$

#### Electron paramagnetic resonance spectroscopy

Electron paramagnetic resonance spectra were acquired using a Miniscope MS300 X-band spectrometer (Magnettech, Germany). A 50 μL aliquot of the mixture containing 1 mg·mL^−1^ of g-C_3_N_4_ sample and 0.05 mmol·L^−1^ DMPO in DMSO-water mixed solvent 4:1 (v/v) was briefly sonicated and then aspired into a capillary. The EPR spectra were measured in the dark and then directly irradiated in the EPR cavity. Ten spectra were recorded in sequence, with an acquisition time of 30 s, modulation amplitude of 0.2 mT, and microwave attenuation factor set at 10 dB. A Lightningcure LC8 (Hamamatsu, Japan), featuring a high-intensity mercury-xenon lamp set at 100% intensity, served as the light source, and the far-UV part of the spectrum was removed using an A9616-05 filter (Hamamatsu, Japan).

The EPR spectra were quantitatively assessed by converting the EPR double integrals to spin concentration values. For this purpose, the EPR spectrum of TEMPOL standard stable radical TEMPOL was recorded (in the absence of illumination under otherwise identical instrument settings), baseline-corrected, and double-integrated to obtain the conversion factor. The obtained spin concentrations were normalised per unit catalyst mass and used to calculate the initial rates of spin adduct formation.

#### Photocatalytic experiments

All chemicals used were of analytical reagent grade (*pro analysi*, purity 99.0–99.8%). Deionised water with a pH of 6.5–6.9 and conductivity of <0.1 μS·cm^-1^ (AQUAL 29, Czech Republic) was used throughout the study.

Photocatalytic degradation experiments were performed in suspensions containing 10 mg of bulk or exfoliated g-C_3_N_4_ photocatalysts in 150 mL aqueous solutions of ofloxacin (c = 10 mg·L^−1^), diclofenac, or caffeine (c = 20 mg·L^−1^). The suspensions were stirred in the dark for 60 min to reach adsorption-desorption equilibrium and then irradiated with an LED source (420 nm, intensity of 13.5 mW cm^−2^) for 120 min. The temperature of the reaction mixture was maintained at 20 °C. Aliquots (3 mL) were collected at regular intervals and filtered using Chromafil GF/RC-20/25 syringe filters (pore size 0.2–1.0 μm).

The filtrates were analyzed using a high-performance liquid chromatography (HPLC) Nexera XR chromatograph (Shimadzu, Kyoto, Japan) equipped with a photodiode array (PDA) detector. An analytical Kinetex Biphenyl 100 Å column (150 mm length, 4.6 mm i.d., 2.6 μm core-shell particles; Phenomenex, Torrance, CA, USA) was used for HPLC analysis. HPLC separations were performed in isocratic mode, with a mobile phase consisting of 0.1% formic acid in ultrapure water and a mixture of acetonitrile and methanol (85:15, v/v) in a ratio of 40:60 (v/v). The analysis time was 5 min, the flow rate was 1 mL·min^−1^ and the injection volume was 10 μL.

#### Identification of intermediary transformation products and prediction their toxicity

To identify intermediary transformation products, liquid chromatography combined with high-resolution tandem mass spectrometry was employed. For this purpose, 10 mL aliquots were collected before and after the termination of the photocatalysis process and filtered. Blank (demineralised water in contact with g-C_3_N_4_ particles for 1 h) and standard samples (stock solutions of the pharmaceuticals diluted with demineralised water at appropriate concentrations) were prepared for comparative purposes.

HPLC/HRTMS analysis was performed using an Acquity HPLC system coupled to a high-resolution tandem mass spectrometer (Synapt G2-S; Waters, Massachusetts, USA). The same Kinetex Biphenyl 100 Å column (150 mm length, 4.6 mm i.d., 2.6 μm core-shell particles; Phenomenex, Torrance, CA, USA) was used for chromatographic separation, along with a mobile phase consisting of ultrapure water (A) and acetonitrile (B), both acidified with 0.1% formic acid. A gradient method was applied with a profile as follows: start with 5% B, followed by an increase to 90% B in 1 min, held for 5 min, decreased to 5% B in 20 s, and then held for 2 min and 40 s. The injection volume was 10 μL, and the flow rate was 0.5 mL min^−1^. The parameters of the mass spectrometer were as follows: spray voltage of +2.5 kV, sample cone voltage of 18 V, source temperature of 100 °C, desolvation temperature of 250°C, desolvation gas flow of 600 L/h, analyser in V-mode, scan range of 50–1200 (Da), scan time of 0.2 s, and interscan delay of 0.02 s. MS/MS data were obtained via fragmentation experiments using a trap-collision cell. A collision energy of 25 eV was used. The instrument was calibrated using a sodium formate solution (0.5 mmol L^−1^, dissolved in 90:10 2-propanol/water, v/v). Leucine-enkephalin (50 pg μL^−1^, dissolved in 50:50 acetonitrile/water + 0.1% formic acid, v/v/v) was used for lock mass correction using a reference electrospray probe during an HPLC/HRTMS run. Data collection was performed using MassLynx software (version 4.1; Waters, Milford, MA, USA).

The quantitative structure–activity relationship method was used to predict the ecotoxicity of the identified TPs. The freely available software QSAR Toolbox by OECD and ECHA was used for this purpose, implying a method previously described in the literature.^59^

### Quantification and statistical analysis

Confidence intervals were calculated for *n* = 3–5 replicates at the significance level α = 0.05.

## Supplemental information

Document S1. Figures S1–S13, Tables S1–S7, and supplemental references
