# Supplementary material for: Photocatalytic degradation and transformation of pharmaceuticals using exfoliated metal-free g-C3N4
Source: iScience. 2025 Oct 30;28(12):113899. doi: 10.1016/j.isci.2025.113899 (PMC12682146; doi:10.1016/j.isci.2025.113899)
Supplement: Document S1. Figures S1–S13, Tables S1–S7, and supplemental references [file mmc1.pdf]

iScience, Volume *28*

Supplemental information

Photocatalytic degradation and transformation of pharmaceuticals using exfoliated metal-free g-C_3N_4

Petr Praus, Anna Gavlová, Jan Hrbáč, Kristina Schmidtová, and Petr Bednář

Supplemental Information

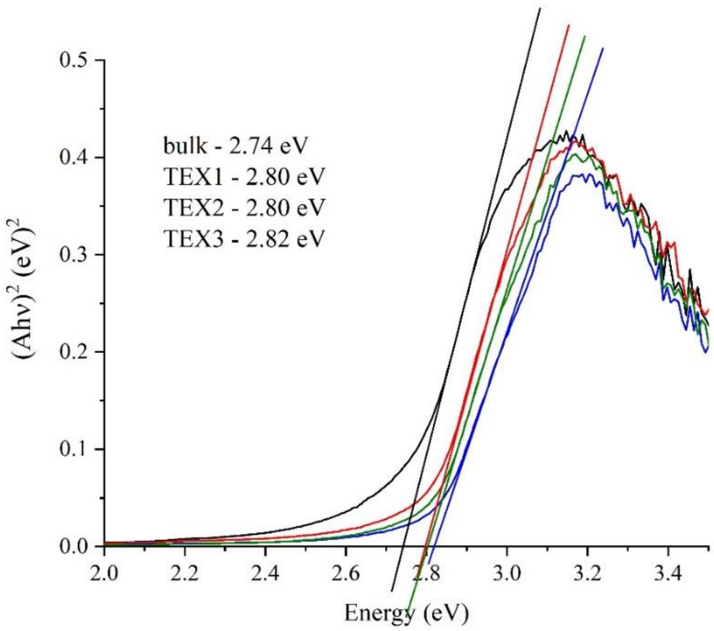

Figure S1. Determination of band gap energies of bulk and exfoliated graphitic carbon nitride according to Tauc´s approach.

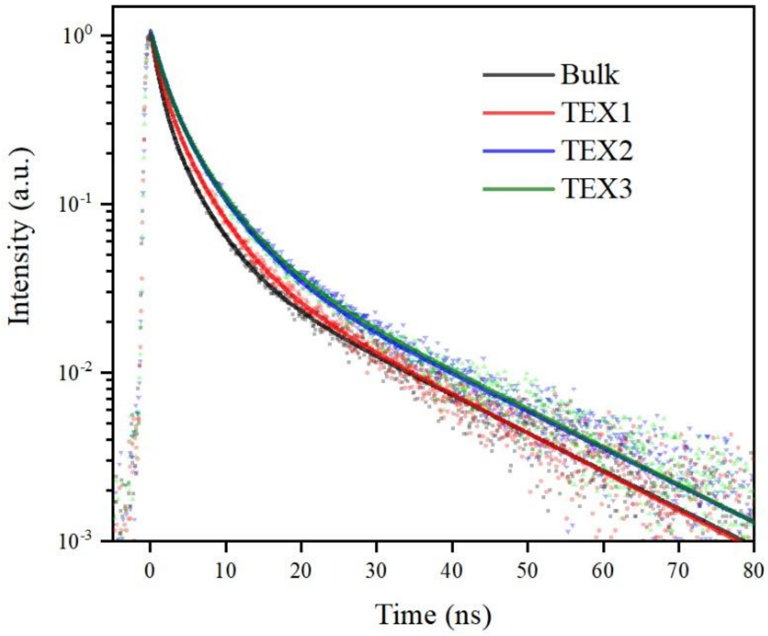

Figure S2. Photoluminescence decay curves of bulk and exfoliated graphitic carbon nitride. An EPL-375 ps pulsed diode laser (λ_{em} = 372 nm) with a pulse width of 66.5 ps, repetition rate of 20 MHz, and average power of 75 μW was used.

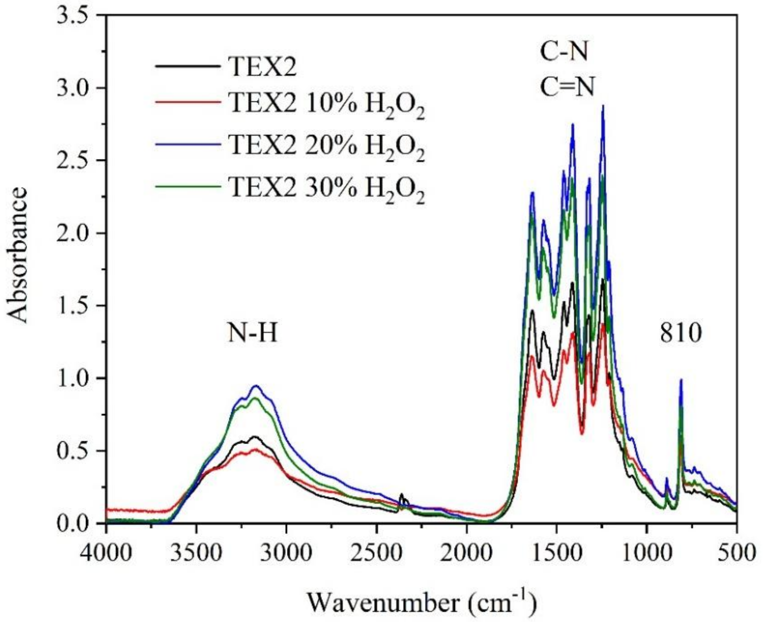

Figure S3. FTIR spectra of TEX2 treated in 10-30 % hydrogen peroxide solutions for 5 h with a resolution of 2 cm^{-1}. The KBr method was employed.

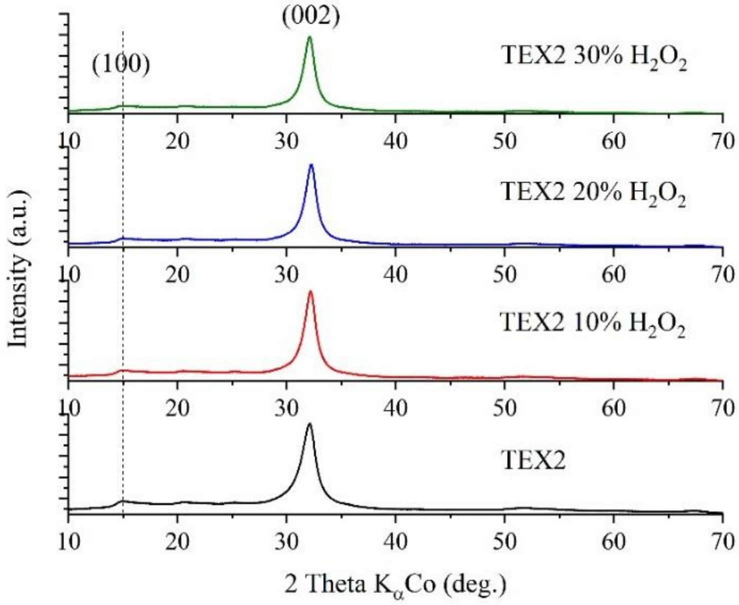

Figure S4. XRD patterns (Co tube) of TEX2 treated with 10-30 % hydrogen peroxide solutions for 5 h. The (002) peaks were shifted from 32.1-32.2 of 2 Theta degrees.

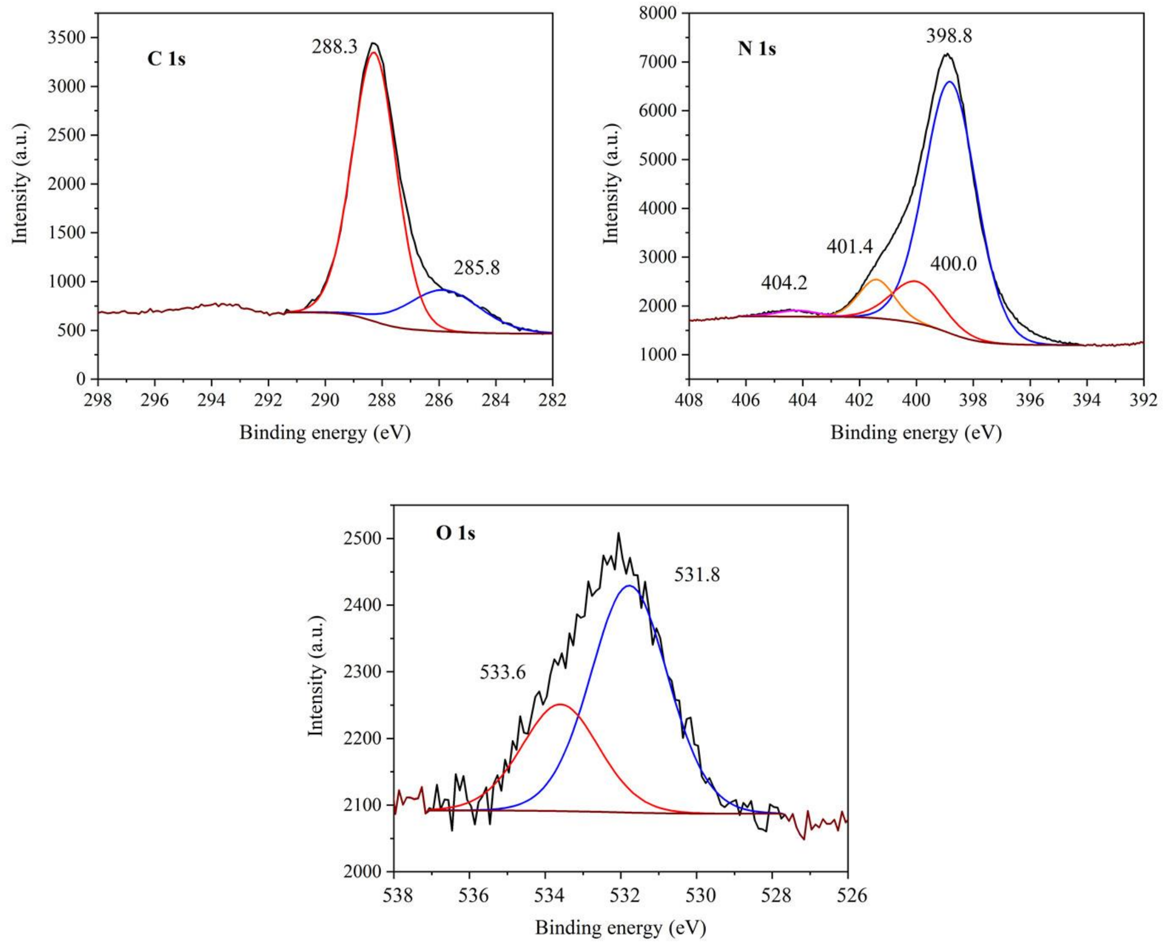

Figure S5. XPS spectra of TEX3 treated in 30% hydrogen peroxide using Mg Kα radiation (hν = 1253.6 eV) generated at 12 kV and 10 mA.

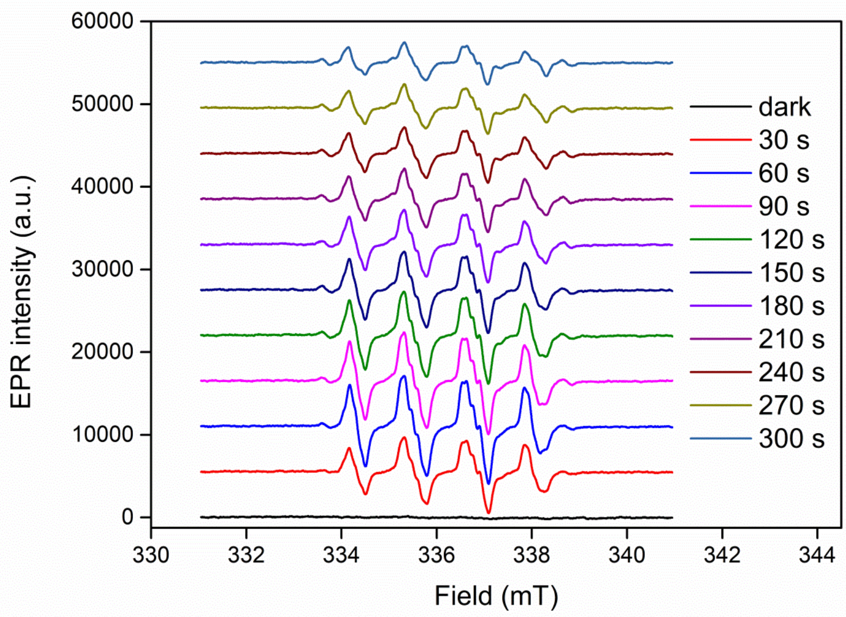

Figure S6. Set of EPR spectra obtained from the DMPO spin trapping experiment with a bulk graphitic carbon nitride sample. The acquisition time of each spectrum was 30 s with a modulation amplitude of 0.2 mT and a microwave attenuation factor of 10 dB.

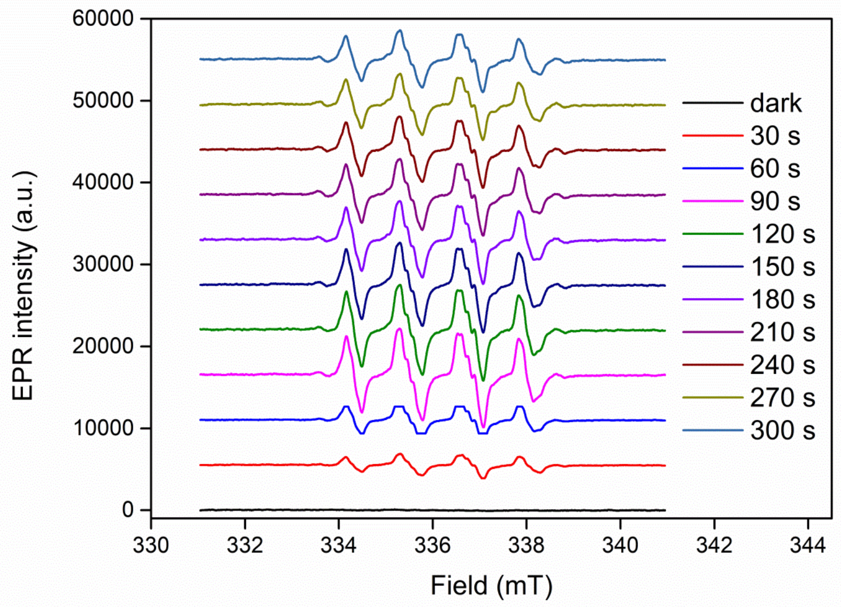

Figure S7. Set of EPR spectra for the TEX1 sample. The acquisition time of each spectrum was 30 s with a modulation amplitude of 0.2 mT and a microwave attenuation factor of 10 dB.

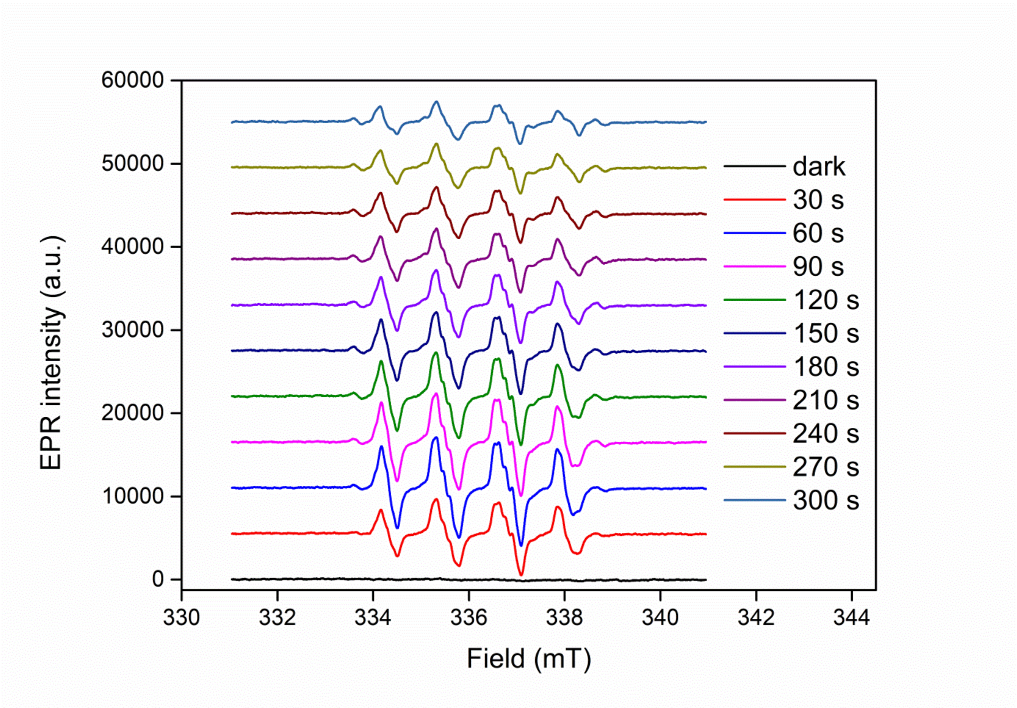

Figure S8. Set of EPR spectra for the TEX3 sample. The acquisition time of each spectrum was 30 s with a modulation amplitude of 0.2 mT and a microwave attenuation factor of 10 dB.

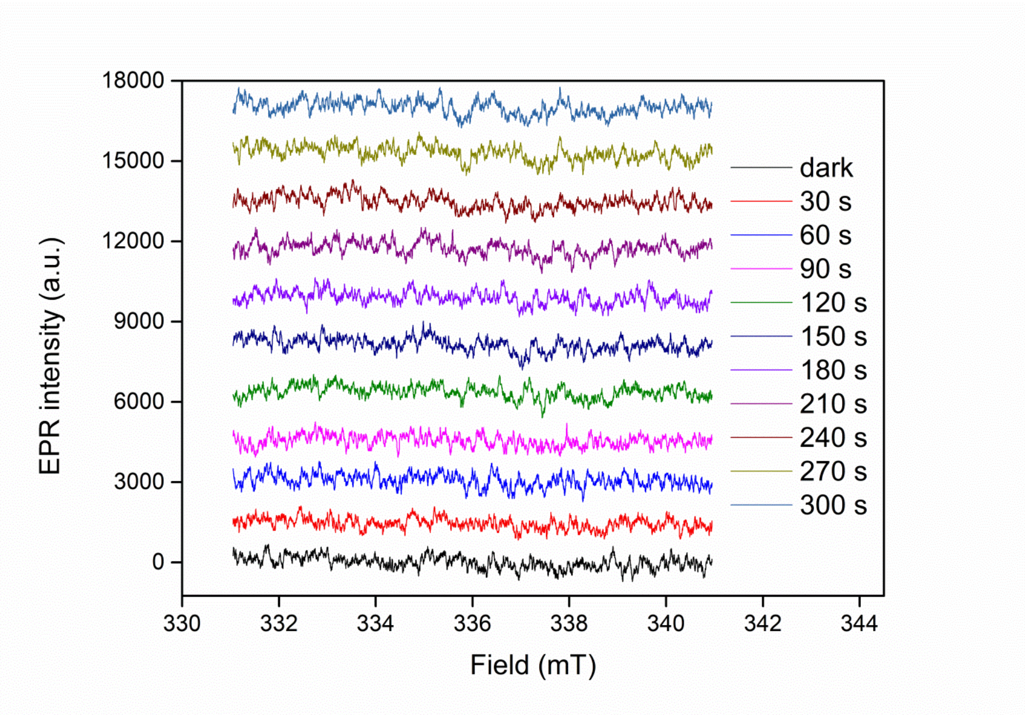

Figure S9. Set of EPR spectra from a control experiment involving irradiation of the DMPO spin trap in the absence of graphitic carbon nitride. The acquisition time of each spectrum was 30 s with a modulation amplitude of 0.2 mT and a microwave attenuation factor of 10 dB.

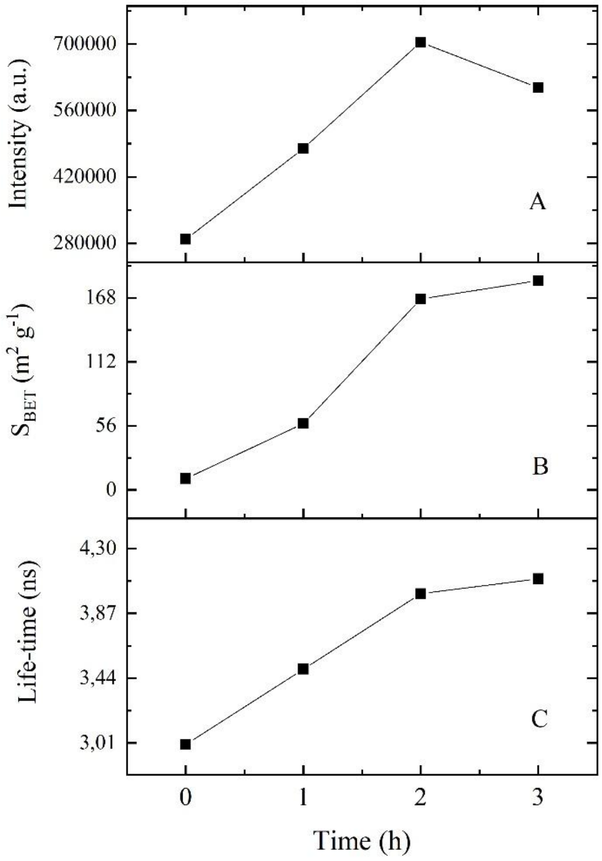

Figure S10. Comparison of (A) the maximal PL intensities, (B) specific surface area, and (C) average lifetimes (for two components) of graphitic carbon nitride depending on the exfoliation time.

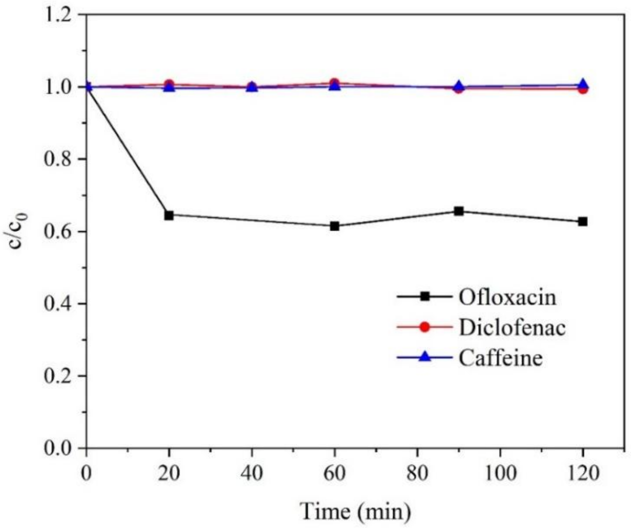

Figure S11. Kinetic curves of the photocatalytic degradation of ofloxacin, diclofenac, and caffeine using TEX3 photocatalysts in the absence of irradiation.

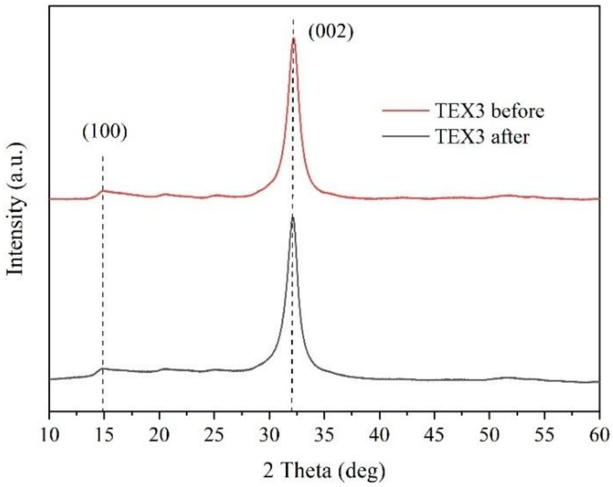

Figure S12. X-ray diffraction patterns (Co tube) of TEX3 before and after 3cycles of the photocatalytic degradation of ofloxacin.

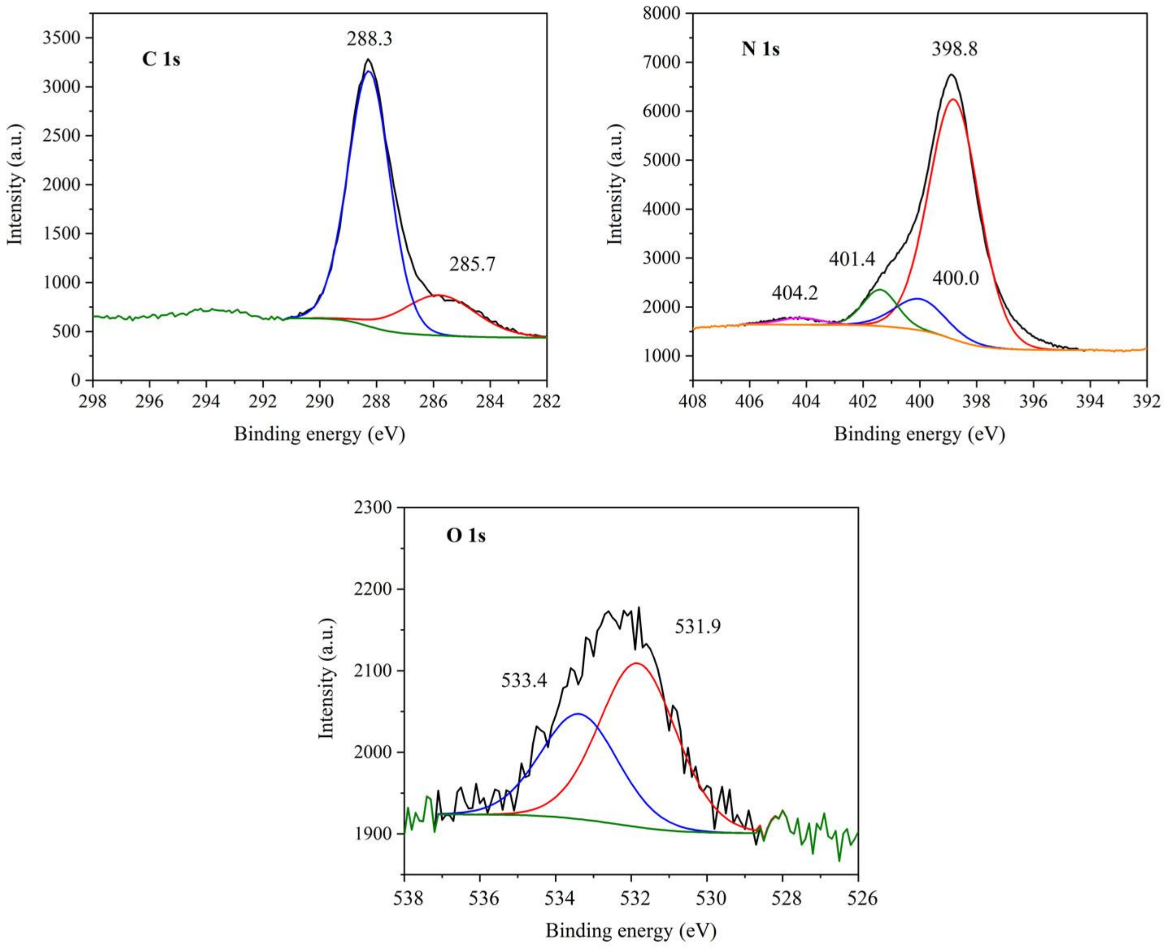

Figure S13. XPS spectra of TEX3 after 3 cycles of the photocatalytic degradation of ofloxacin using Mg Kα radiation (hν = 1253.6 eV) generated at 12 kV and 10 mA.

Table S1. SEM-EDS analysis of bulk and exfoliated g-C_3N_4

**Material**	**C (wt.%)**	**N (wt.%)**	**C/N**	**O (wt.%)**
Bulk	45.80	51.87	0.883	2.33
TEX1	51.01	46.11	1.11	2.88
TEX2	51.06	46.23	1.10	2.71
TEX3	47.36	50.30	0.942	2.34

Table S2. XPS analysis of bulk and TEX3 g-C_3N_4

**Material**	**C 1s (at. %)**	**O 1s (at. %)**	**N 1s (at. %)**	**C/N**
Bulk	46.16	3.03	50.81	0.908
TEX3	42.94	1.23	55.83	0.769
TEX3 10% H_2O_2	42.77	2.10	55.13	0.776
TEX3 30% H_2O_2	42.22	2.84	54.94	0.768

Table S3. Photocatalytic degradation of ofloxacin, diclofenac, and caffeine using various photocatalysts

**Photocatalyst**	**Pharmaceutical**	**k x 10^{-3} (min^{-1})**	**Efficiency**	**Reference**
$BiFeO_3$	Ofloxacin	9.70	64% in 180 min	[1]
Bi_2WO_6/Au NPs	Ofloxacin	24.5	95% in 180 min	[2]
CdS/TiO_2	Ofloxacin	8.96	86% in 180 min	[3]
$MnWO_4$/g-C_3N_4	Ofloxacin	33.4	90.4% in 70 min	[4]
La/TiO_2	Diclofenac	7.05	98% in 240 min	[5]
Fe_2O_3	Diclofenac	40.0	96% in 120 min	[6]
La/$Cu_{0.6}Cd_{0.4}S$	Diclofenac	36.0	89% in 240 min	[7]
g-C_3N_4/C QDs	Diclofenac	20.0	98% in 150 min	[8]
TiO_2 film	Caffeine	12.1	99% in 180 min	[9]
$CsBa_2Ta_3O_{10}$	Caffeine	2.81	60% in 60 min	[10]
ZrO_2	Caffeine	0.831	21% in 240 min	[11]
WS_2/Graphene	Caffeine	10.8	93% in 180 min	[12]
Exfoliated g-C_3N_4 (TEX2)	Ofloxacin	27.5 ± 2.3	95% in 120 min	This work
Exfoliated g-C_3N_4 (TEX2)	Diclofenac	28.7 ± 2.6	95% in 120 min	This work
Exfoliated g-C_3N_4 (TEX2)	Caffeine	12.6 ± 0.9	80% in 120 min	This work

Note: k is the rate constant; NPs – nanoparticles; QDs – quantum dots, GO – graphene oxide.

Table S4. LC-MS data for ofloxacin and its transformation products

Structure	Compound	Formula	m/z experiment.	m/z theoretical	Error (ppm)	Ret. time (min)
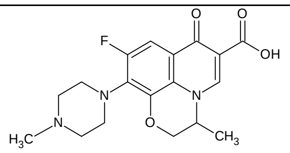	Ofloxacin	$C_{18}H_{20}FN_3O_4$	362.1486	362.15106	-6.79	5.99
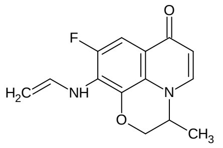	TP 1	$C_{14}H_{13}FN_2O_2$	261.1034	261.10338	0.08	4.84
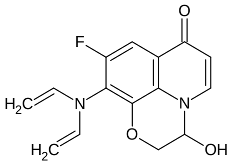	TP 2	$C_{15}H_{13}FN_2O_3$	289.0884	289.09829	-34.23	6.82
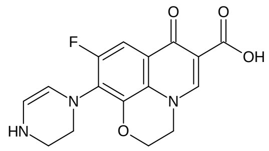	TP 3	$C_{16}H_{14}FN_3O_4$	332.1091	332.10410	15.05	5.47
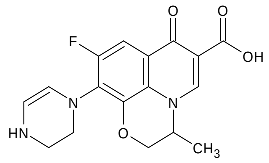	TP 4	$C_{17}H_{16}FN_3O_4$	346.1216	346.11976	5.31	5.76
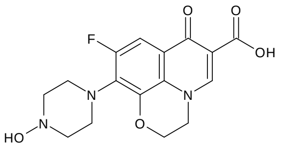	TP 5a	$C_{16}H_{16}FN_3O_5$	350.1143	350.11467	-1.05	5.51
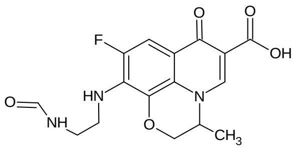	TP 5b	$C_{16}H_{16}FN_3O_5$	350.1143	350.11467	-1.05	5.51
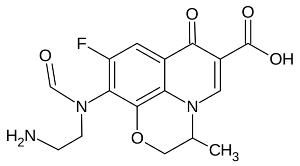	TP 5c	$C_{16}H_{16}FN_3O_5$	350.1143	350.11467	-1.05	5.51

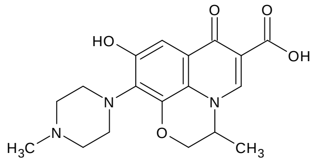	TP 6	$C_{18}H_{21}N_3O_5$	360.1345	360.15539	-58.00	6.57
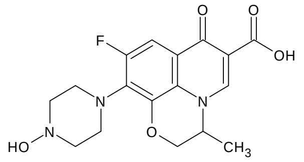	TP 7a	$C_{17}H_{18}FN_3O_5$	364.1278	364.13032	-6.92	5.61
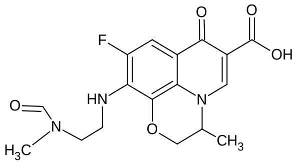	TP 7b	$C_{17}H_{18}FN_3O_5$	364.1278	364.13032	-6.92	5.61
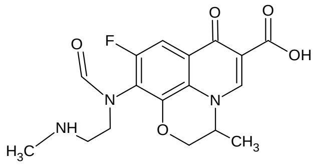	TP 7c	$C_{17}H_{18}FN_3O_5$	364.1278	364.13032	-6.92	5.61
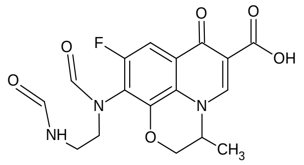	TP 8	$C_{17}H_{16}FN_3O_6$	378.1097	378.10959	0.29	6.58
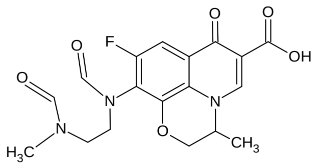	TP 9	$C_{18}H_{18}FN_3O_6$	392.1231	392.12524	-5.45	6.91

Note: Error: mass error; Ret. time – retention time.

Table S5. Caffeine and identified transformation products

Structure	Compound	Formula	m/z experiment.	m/z theoretical	Error (ppm)	Ret. time (min)
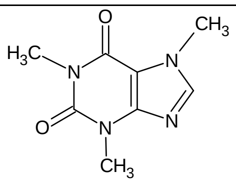	Caffeine	$C_8H_{10}N_4O_2$	195.0908	195.08765	16.14	5.87
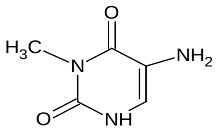	TP 10a	$C_5H_7N_3O_2$	142.0642	142.0611	21.82	4.57
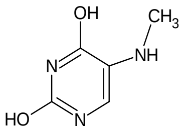	TP 10b	$C_5H_7N_3O_2$	142.0642	142.0611	21.82	4.57

Note: Error: mass error; Ret. time – retention time.

Table S6. Diclofenac and identified transformation products

Structure	Compound	Formula	m/z experiment.	m/z theoretical	Error (ppm)	Ret. time (min)
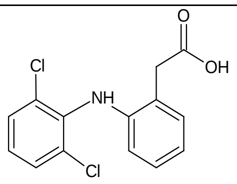	Diclofenac	$C_{14}H_{11}Cl_2NO_2$	296.0224	296.02396	-5.26	8.33
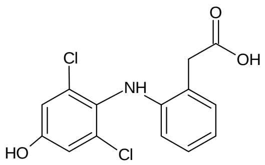	TP 11	$C_{14}H_{11}Cl_2NO_3$	312.0197	312.01887	2.66	7.45
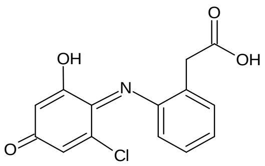	TP 12	$C_{14}H_{10}Cl_2$NO4	291.9893	292.03711	-163.71	7.63

Note: Error: mass error; Ret. time – retention time.

Table S7. The card JCPDS 87-1526 of graphitic carbon nitride

PDF Card No.: 01-087-1526 Quality:B

Sub-File Name: Inorganic, Alloy&Metal, Explosive, ICSD Pattern

Formula: C3 N4

Name: tricarbon tetranitride I/Ic (RIR)= 2.62

Crystal System: Hexagonal Space Group: P-6m2(187) Dmeas:

Cell Parameters: a= 4.7420 b= 4.7420 c= 6.7205

Alpha= 90.000 Beta= 90.000 Gamma= 120.000

Volume= 130.875 Z= 2

Reference: Teter, D.M., Hemley, R.J. Science271(1996)53.

Radiation: CuKalpha1 Wavelength= 1.54060

2Theta range: 13.16 - 149.80

Database comments: ANX: A3X4. ICSD Collection Code: 83265. Hypothetical Structure: Structure calculated theoretically. Minor Warning: No e.s.d reported/abstracted on the cell dimension. Unit Cell Data Source: Powder Diffraction.

Relative Intensity

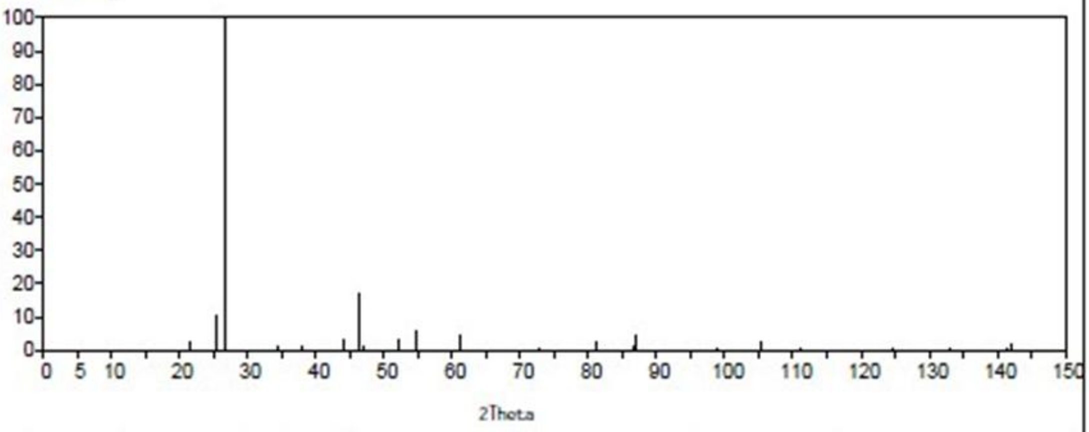

No.	2Theta	d-Value	Intensity	h	k	l	No.	2Theta	d-Value	Intensity	h	k	l
1	13.16	6.720	0.1	0	0	1	21	74.17	1.277	0.1	1	0	5
2	21.62	4.107	2.7	1	0	0	22	74.28	1.276	0.2	2	1	3
3	25.40	3.504	10.3	1	0	1	23	74.83	1.268	0.2	3	0	2
4	26.50	3.360	100.0	0	0	2	24	81.06	1.186	2.8	2	2	0
5	34.46	2.601	1.1	1	0	2	25	85.01	1.140	0.1	2	1	4
6	37.92	2.371	1.2	1	1	0	26	86.46	1.125	1.0	2	0	5
7	40.30	2.236	0.1	0	0	3	27	86.62	1.123	0.2	3	1	1
8	44.07	2.053	3.4	2	0	0	28	86.90	1.120	0.9	0	0	6
9	46.19	1.964	17.0	1	0	3	29	87.11	1.118	4.3	2	2	2
10	46.86	1.937	1.0	1	1	2	30	90.93	1.081	0.1	1	0	6
11	52.16	1.752	3.2	2	0	2	31	91.14	1.079	0.1	3	1	2
12	54.58	1.680	5.7	0	0	4	32	93.08	1.061	0.1	3	0	4
13	59.39	1.555	0.1	1	0	4	33	97.23	1.027	0.1	4	0	0
14	59.51	1.552	0.1	2	1	0	34	98.75	1.015	0.7	3	1	3
15	61.18	1.514	4.7	2	0	3	35	99.03	1.013	0.1	1	1	6
16	66.28	1.409	0.1	2	1	2	36	103.14	0.983	0.2	2	0	6
17	68.38	1.371	0.2	1	1	4	37	103.35	0.982	0.2	4	0	2
18	68.49	1.369	0.1	3	0	0	38	106.35	0.969	2.4	2	2	4
19	70.10	1.341	0.1	3	0	1	39	109.69	0.942	0.1	3	1	4
20	72.65	1.300	0.7	2	0	4	40	110.97	0.935	0.1	1	0	7

Note: 2theta are calculated with wavelength = 1.54059

2016-Apr-18 09:32:26 Page-1/2

PDF Card No.: 01-087-1526 Quality:B

No.	2Theta	d-Value	Intensity	h	k	l
41	111.24	0.933	0.6	4	0	3
42	116.01	0.908	0.1	2	1	6
43	116.23	0.907	0.1	3	2	2
44	118.53	0.896	0.1	4	1	0
45	120.08	0.889	0.1	2	2	5
46	120.26	0.888	0.1	4	1	1
47	123.11	0.876	0.1	4	0	4
48	124.68	0.870	0.4	2	0	7
49	124.99	0.868	0.2	3	1	5
50	125.39	0.867	0.1	3	0	6
51	125.65	0.866	0.1	4	1	2
52	132.97	0.840	0.3	0	0	8
53	138.76	0.823	0.1	1	0	8
54	139.23	0.822	0.1	3	2	4
55	141.51	0.816	0.4	4	0	5
56	142.21	0.814	1.7	2	2	6
57	149.39	0.799	0.1	3	1	6
58	149.80	0.798	0.1	5	0	2

Note: 2theta are calculated with wavelength = 1.54059

2016-Apr-18 09:32:26 Page-2/2

References

[1] G. Gupta, S.K. Kansal, A. Umar, S. Akbar, Visible-light driven excellent photocatalytic degradation of ofloxacin antibiotic using $BiFeO_3$ nanoparticles, Chemosphere, 314 (2023) 137611.

[2] Y. Tian, L. Ma, X. Tian, Y. Nie, C. Yang, Y. Li, L. Lu, Z. Zhou, More reactive oxygen species generation facilitated by highly dispersed bimodal gold nanoparticle on the surface of Bi_2WO_6 for enhanced photocatalytic degradation of ofloxacin in water, Chemosphere, 269 (2021) 128717.

[3] A. Kaur, A. Umar, W.A. Anderson, S.K. Kansal, Facile synthesis of CdS/TiO_2 nanocomposite and their catalytic activity for ofloxacin degradation under visible illumination, Journal of Photochemistry and Photobiology A: Chemistry, 360 (2018) 34-43.

[4] S.L. Prabavathi, K. Saravanakumar, M. Gcina, V. Muthuraj, 1D/2D $MnWO_4$ nanorods anchored on g-C_3N_4 nanosheets for enhanced photocatalytic degradation ofloxacin under visible light irradiation, Colloids and Surfaces A: Physicochemical and Engineering Aspects, 581 (2019) 123845.

[5] H. Lee, J. Park, S.S. Lam, Y.-K. Park, S.-C. Kim, S.-C. Jung, Diclofenac degradation properties of a La-doped visible light-responsive TiO_2 photocatalyst, Sustainable Chemistry and Pharmacy, 25 (2022) 100564.

[6] I. Mimouni, A. Bouziani, Y. Naciri, M. Boujnah, M.A. El Belghiti, M. El Azzouzi, Effect of heat treatment on the photocatalytic activity of α-Fe_2O_3 nanoparticles: towards diclofenac elimination, Environmental Science and Pollution Research, 29 (2022) 7984-7996.

[7] H. Boukhatem, H. Khalaf, L. Djouadi, Z. Marin, R.M. Navarro, J.A. Santaballa, M. Canle, Diclofenac degradation using mont-La (6%)-$Cu_{0.6}Cd_{0.4}S$ as photocatalyst under NUV–Vis irradiation. Operational parameters, kinetics and mechanism, Journal of Environmental Chemical Engineering, 5 (2017) 5636-5644.

[8] Q. You, Q. Zhang, M. Gu, R. Du, P. Chen, J. Huang, Y. Wang, S. Deng, G. Yu, Self-assembled graphitic carbon nitride regulated by carbon quantum dots with optimized electronic band structure for enhanced photocatalytic degradation of diclofenac, Chemical Engineering Journal, 431 (2022) 133927.

[9] R. Muangmora, P. Kemacheevakul, P. Punyapalakul, S. Chuangchote, Enhanced Photocatalytic Degradation of Caffeine Using Titanium Dioxide Photocatalyst Immobilized on Circular Glass Sheets under Ultraviolet C Irradiation, Catalysts, 10 (2020) 964.

[10] B. Czech, M. Hojamberdiev, UVA- and visible-light-driven photocatalytic activity of three-layer perovskite Dion-Jacobson phase $CsBa_2M_3O_{10}$ (M=Ta, Nb) and oxynitride crystals

in the removal of caffeine from model wastewater, Journal of Photochemistry and Photobiology A: Chemistry, 324 (2016) 70-80.

[11] M.G. Verma, R.S. Das, A. Kumar, Cu–ZrO_2@GO scaffold: Visible spectrum triggered caffeine degradation and microbial inactivation, Ceramics International, 50 (2024) 34623-34638.

[12] T. Shafi, C. Das, B.K. Dubey, S. Chowdhury, Aerogels Made of Few-Layer WS_2 Nanosheets and Nitrogen-Doped Graphene for Photocatalytic Degradation of Caffeine, ACS Applied Nano Materials, 7 (2024) 1723-1737.
